# Drug-Loaded Mesoporous Polydopamine Nanoparticles in Chitosan Hydrogels Enable Myocardial Infarction Repair through ROS Scavenging and Inhibition of Apoptosis

**DOI:** 10.1021/acsami.4c08155

**Published:** 2024-09-30

**Authors:** Tianhu Wang, Yabin Wang, Yingjie Zhang, Zhiyi Fang, Sulei Li, Zhenghui Gu, Yan Ma, Linghuan Wang, Dong Han, Changyong Wang, Jin Zhou, Feng Cao

**Affiliations:** † Chinese PLA Medical School & Department of Cardiology, The Second Medical Center National Clinical Research Center for Geriatric Diseases, Chinese PLA General Hospital, Beijing 100853, China; ‡ School of Medicine, Nankai University, Tianjin 300071, China; § Beijing Institute of Basic Medical Sciences, Beijing 100850, China

**Keywords:** chitosan, injectable hydrogel, myocardial infarction, EGCG, PDA

## Abstract

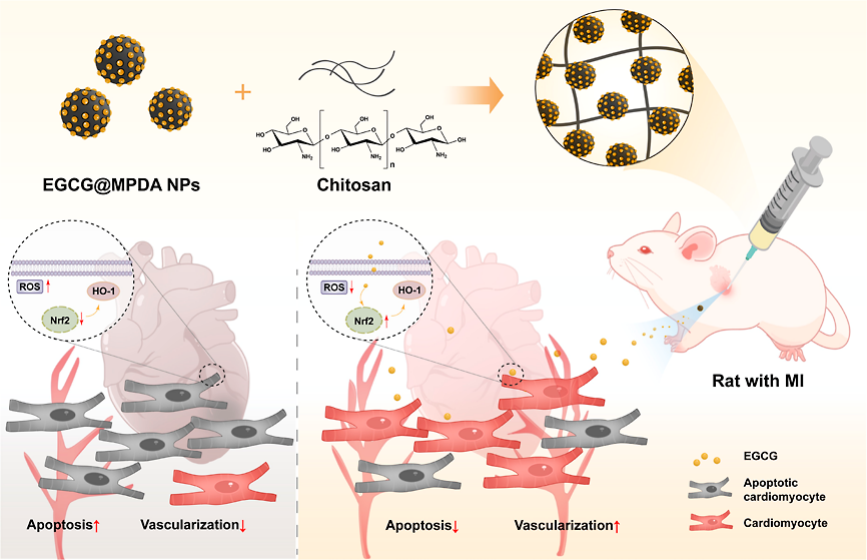

In this study, we synthesized mesoporous polydopamine nanoparticles (MPDA NPs) using an emulsion-induced interface assembly strategy and loaded epigallocatechin gallate (EGCG) into MPDA NPs via electrostatic attraction to form EGCG@MPDA NPs. In the post myocardial infarction (MI) environment, these interventions specifically aimed to eliminate reactive oxygen species (ROS) and facilitate the repair of MI. We further combined them with a thermosensitive chitosan (CS) hydrogel to construct an injectable composite hydrogel (EGCG@MPDA/CS hydrogel). Utilizing *in vitro* experiments, the EGCG@MPDA/CS hydrogel exhibited excellent ROS-scavenging ability of H9C2 cells under the oxidative stress environment and also could inhibit their apoptosis. The EGCG@MPDA/CS hydrogel significantly promoted left ventricular ejection fraction (LVEF) in infarcted rat models post injection for 28 days compared to the PBS group (51.25 ± 1.73% vs 29.31 ± 0.78%, *P* < 0.05). In comparison to the PBS group, histological analysis revealed a substantial increase in left ventricular (LV) wall thickness in the EGCG@MPDA/CS hydrogel group (from 0.58 ± 0.03 to 1.39 ± 1.11 mm, *P* < 0.05). This work presents a novel approach to enhance MI repair by employing the EGCG@MPDA/CS hydrogel. This hydrogel effectively reduces local oxidative stress by ROS and stimulates the nuclear factor erythroid 2-related factor 2 (Nrf2)/heme oxygenase-1 (HO-1) pathway.

## 1 Introduction

At a global scale, cardiovascular disease is the primary factor responsible for both death and disability. Caused by blockage of coronary arteries, myocardial infarction (MI) results in permanent damage and death of myocardial cells.^1^ Revascularization, including percutaneous coronary intervention (PCI), thrombolysis, and coronary artery bypass grafting, is the main emphasis of modern medical interventions for acute MI treatment.^2,3^ However, the infarcted myocardium cannot regenerate, ultimately leading to end-stage heart failure. Various pathological changes occur following acute MI, including oxidative stress, cell apoptosis, and the release of inflammatory factors. These ultimately result in extensive loss of myocardial cells,^4,5^ impaired cardiac function, and ventricular remodeling. Intense generation of reactive oxygen species (ROS) during MI surpasses the cellular antioxidant capacity, leading to oxidative stress.^6,7^ The problem in eliminating ROS after MI is the insufficient retention of pharmaceuticals at the site of the infarction caused by the fast blood flow in the heart. This leads to inefficient treatment with current anti-inflammatory and antioxidant medications.^8^

Tissue engineering and regenerative medicine present encouraging approaches for heart repair following MI. Hydrogels are classes of networked structures with a three-dimensional swelling structure which possess excellent biocompatibility, an internal porous structure, and tunable mechanical strength.^9^ Injectable hydrogels based on natural polysaccharides have shown significant potential in MI repair.^10,11^ Chitosan is a naturally occurring cationic linear polysaccharide composed of repeating units of *N*-acetyl-d-glucosamine. Synthesized through the deacetylation of chitin, it can be enzymatically degraded by lysozymes within the human body.^12^ Demonstrating exceptional biocompatibility and degradability, it finds extensive application in the fabrication of natural hydrogels.^13^

Despite the proven efficacy of specific natural product derivatives and synthesized nanomaterials, such as melanin/alginate hydrogels, iron-curcumin nanozymes, and molybdenum-based nanodots, in eliminating ROS, they face limitations such as low elimination efficiency, restricted biocompatibility, high cost, and inadequate biodegradability.^14,15^ Polydopamine (PDA) is a polymer synthesized by oxidative polymerization of dopamine, exhibiting excellent cell affinity and tissue compatibility, and is capable of scavenging ROS radicals.^16^ In order to augment its efficiency in scavenging ROS, we contemplated the inclusion of epigallocatechin gallate (EGCG). EGCG, the predominant polyphenolic component found in green tea, has inherent anti-inflammatory and antioxidant characteristics.^17^ When combined with PDA, it can enhance the ROS-scavenging capacity of PDA. Moreover, PDA’s sustained release of EGCG can increase its retention time at the infarct site. However, typical PDA materials have a relatively low drug-loading capacity. Mesoporous polydopamine (MPDA) with a mesoporous structure offers a larger surface area and higher drug-loading capacity, making it an attractive new option for drug loading with PDA materials.

This study synthesized EGCG-loaded mesoporous polydopamine nanoparticles (EGCG@MPDA NPs) encapsulated in chitosan hydrogel for post MI repair. This newly fabricated hydrogel encapsulating EGCG@MPDA NPs has the potential to be a new therapeutic choice for treating MI by eliminating ROS and inflammation, which attenuated cardiomyocyte apoptosis under oxidative stress.

## 2 Results and Discussion

### 2.1 Preparation and Characterization of MPDA NPs and EGCG@MPDA NPs

**Figure 1:**
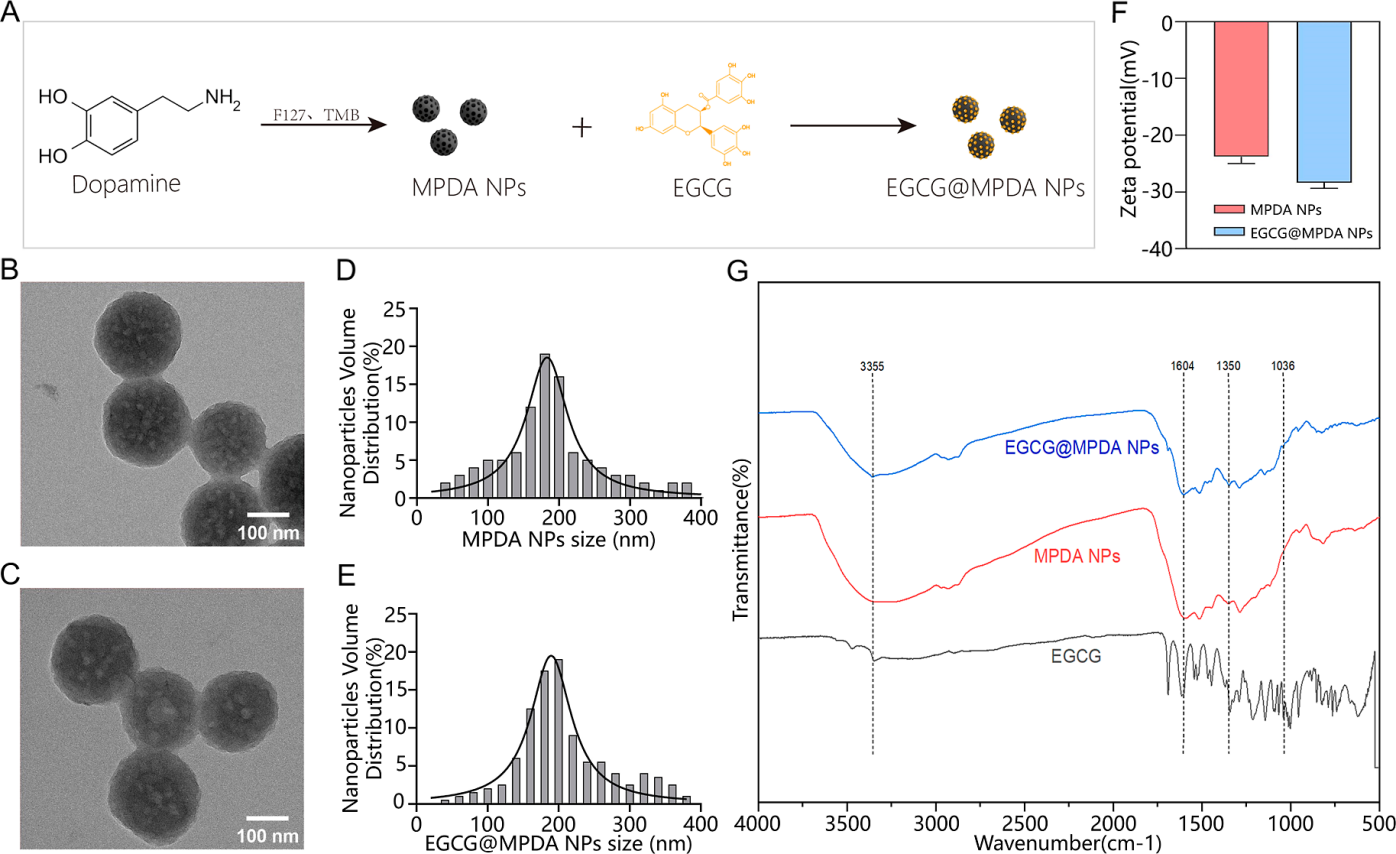
Nanoparticle preparation and characterization. (A) Production of MPDA NPs and EGCG@MPDA NPs, (B,C) TEM images, (D,E) distribution of particle sizes, and (F) zeta potential. (G) FTIR spectra of EGCG, MPDA NPs, and EGCG@MPDA NPs.

The synthesis pathway of EGCG@MPDA NPs is shown in Figure 1A. The synthesis of MPDA nanoparticles was achieved by employing Pluronic F-127 and 1,3,5-trimethylbenzene (TMB) as organic molecules of template within an aqueous solution. Subsequently, EGCG-loaded EGCG@MPDA NPs were further synthesized. Transmission electron microscopy (TEM) revealed that both MPDA NPs and EGCG@MPDA NPs exhibited spherical structures (Figure 1B,C). Compared to MPDA NPs, the surface cavities of EGCG@MPDA NPs became blurred and decreased in quantity, indicating the successful loading of EGCG into the mesoporous structure of MPDA. The distribution of particle sizes of MPDA NPs and EGCG@MPDA NPs was studied through the nanometer particle size potentiometer (Figure 1D,E). The average hydrodynamic size of MPDA NPs was approximately 190.5 nm, which was consistent with previous research,^18^ and the average diameter of EGCG@MPDA NPs was approximately 207.6 nm. MPDA NPs and EGCG@MPDA NPs had exceptional dispersibility and uniformity, as indicated by their polydispersity index (PDI) values below 0.2. Furthermore, the zeta potential of MPDA NPs was determined to be −23.77 ± 1.23 mV, whereas the zeta potential of EGCG@MPDA NPs was seen to be −28.34 ± 1.01 mV (Figure 1F). The changes in zeta potential and particle size indirectly indicate the successful loading of EGCG. Analysis of the materials’ elemental composition and structure was conducted using Fourier transform infrared spectroscopy (FTIR), which also enabled the detection of different intrinsic functional groups in molecules.^19^ To examine the structural modifications of MPDA following interaction with EGCG molecules, we performed an analysis of the FTIR spectra of EGCG, MPDA NPs, and EGCG@MPDA NPs (Figure 1G). Compared to the spectrum of MPDA NPs, only some peaks of EGCG@MPDA NPs showed changes in position and intensity, attributed to specific functional groups. The differences in absorption peaks seen at wavenumbers of 1350, 1604, and 1036 cm^–1^ were attributed to the elongation of C=O bonds, the bending of C–C bonds, and the contraction of O–H bonds, respectively.^20,21^ Moreover, the newly observed absorption peak at 3355 cm^–1^ may be ascribed to the elongation of O–H bonds in hydroxyl groups in EGCG. Overall, the spectra of EGCG@MPDA NPs exhibited minor changes compared to those of MPDA NPs, with characteristic absorption peaks of both EGCG and MPDA NPs present. These results suggested that EGCG is physically coupled to the surface of MPDA NPs via hydrogen bonds, and the incorporation of EGCG did not alter the structure of MPDA NPs. Furthermore, the average drug-loading capacity of EGCG@MPDA NPs was 37.83%, whereas nonporous PDA NPs had a drug-loading capacity of only 7%–10%.^22^ Therefore, MPDA NPs can load more drug because of their mesoporous structure.

### 2.2 Preparation, Characterization, and Biocompatibility Testing of Hydrogels

**Figure 2:**
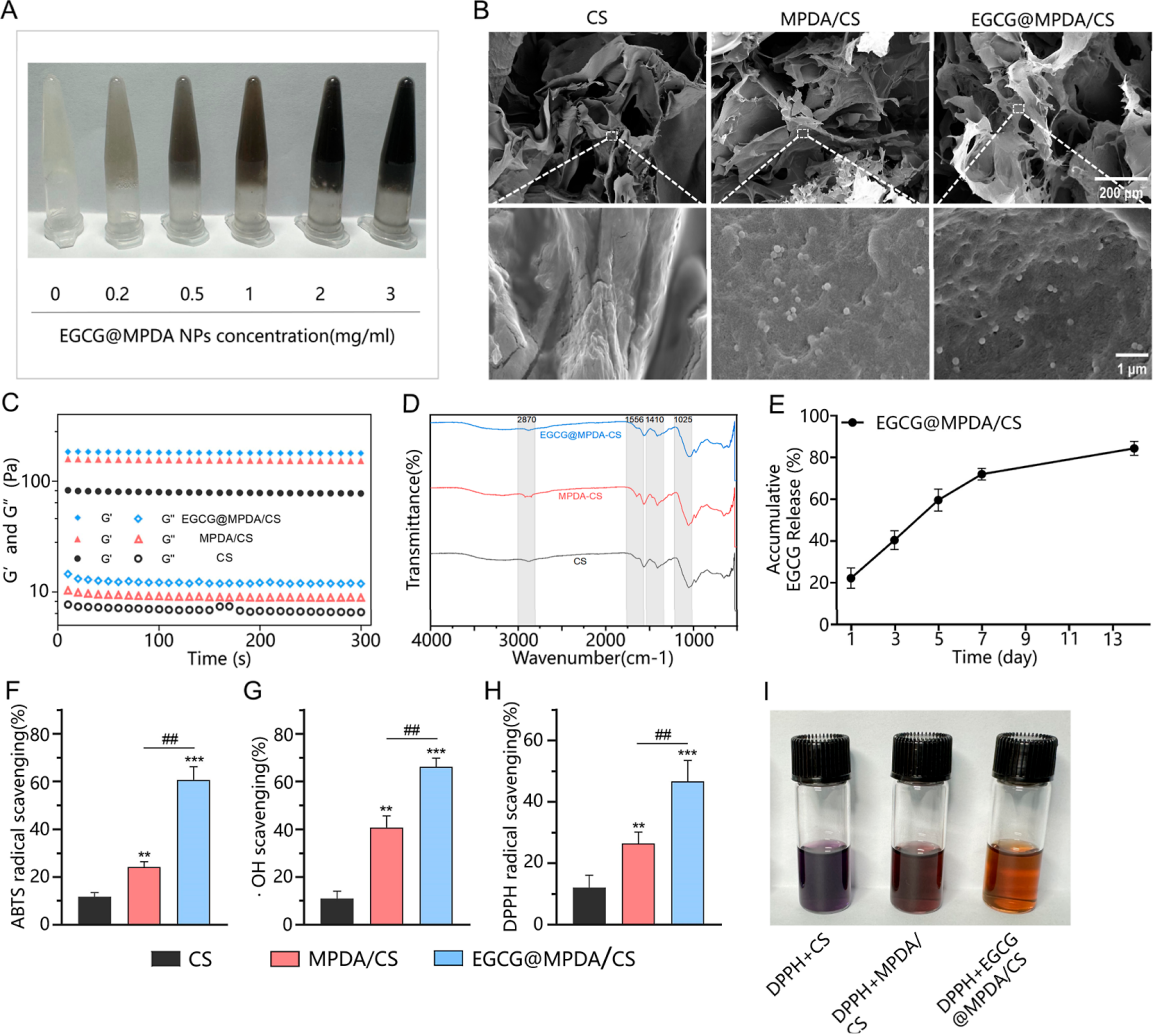
Preparation and characterization of hydrogels. (A) Observation of the formation and stability of EGCG@MPDA/CS hydrogels with different concentrations of EGCG@MPDA NPs after 5 min. (B) SEM images of different hydrogels. (C) Storage modulus (*G*′) and loss modulus (*G*″) of different hydrogels. (D) FTIR spectra of different hydrogels. (E) Cumulative *in vitro* release of EGCG in the EGCG@MPDA/CS hydrogel. Evaluation of the radical-scavenging activity of different hydrogels using the ABTS radical (F), •OH (G), and DPPH radical (H). (I) Optical images of different hydrogels for DPPH radical-scavenging activity. (Results are presented as the mean ± SD; *n* = 6 for each group. **P < 0.01, ***P < 0.001 vs CS group; ^##^*P* < 0.01 vs MPDA/CS group).

MPDA/CS and EGCG@MPDA/CS hydrogels with varying concentrations were efficiently synthesized within 5 min after the introduction of the β-glycerophosphate (β-GP) solution. MPDA NPs and EGCG@MPDA NPs were evenly distributed, leading to homogeneous hydrogel formulations. Chitosan gelation is enhanced by the inclusion of β-GP through three mechanisms: increasing the pH to the physiological range of 7.0–7.4, inhibiting precipitation formation, and imparting thermosensitive properties to the chitosan hydrogel.^23^ As the concentrations of MPDA NPs and EGCG@MPDA NPs increased, the apparent color of the hydrogels deepened gradually (Figure 2A). Through the addition of various concentrations of EGCG@MPDA/CS hydrogels to the medium for cultivating H9C2 cells, we examined the cytocompatibility of the hydrogels. Experiments with the Cell Counting Kit-8 (CCK-8) showed that EGCG@MPDA/CS hydrogels exhibited an exceptional biocompatibility. H9C2 cell viability decreased with the increasing concentration of EGCG@MPDA/CS (Figure S1A). H9C2 cell viability was good when added with the CS hydrogel, 2 mg/mL EGCG@MPDA/CS hydrogel, and 2 mg/mL MPDA/CS hydrogel (Figure S1B). On the seventh day of culturing H9C2 cells, live/dead cell staining showed only a small amount of cell death in the CS hydrogel, 2 mg/mL EGCG@MPDA/CS hydrogel, and 2 mg/mL MPDA/CS hydrogel groups (Figure S2). The potential hemolytic properties of biomaterials intended for clinical use should be evaluated. *In vivo* hemolysis (red blood cell rupture) can result in conditions such as anemia, jaundice, and other pathological manifestations. The hemolysis rate results (Figure S3) indicated that the hemolysis rates of the CS hydrogel group and the 2 mg/mL EGCG@MPDA/CS and MPDA/CS hydrogel groups were all below 5%. This suggests that the prepared hydrogels exhibit excellent blood compatibility.

Images captured with scanning electron microscopy (SEM) showed that MPDA/CS and EGCG@MPDA/CS hydrogels have a porous structure, enabling efficient oxygen and nutrient transfer (Figure 2B). Upon rheological analysis, it was shown that the storage modulus (*G*′) of all hydrogels surpassed the loss modulus (*G*″), providing proof of the effective formation of solid hydrogels (Figure 2C). Furthermore, the inclusion of both types of particles did not have any impact on the formation of chitosan hydrogels. The changes in *G*′ and *G*″ with frequency for the hydrogels are shown in Figure S4A. All the hydrogel materials exhibited *G*′ values that exceeded their respective *G*″ values. This finding indicates that the hydrogels have the ability to continuously maintain their solid state over the spectrum of frequencies that were investigated. Furthermore, the hydrogels were subjected to FTIR analysis to investigate the influence of nanoparticle incorporation on the chemical composition of the hydrogel (Figure 2D). Absorptivity peaks at approximately 2870 cm^–1^ in the CS hydrogel are associated with the stretching vibrations of C–H bonds.^24^ The peaks observed at 1556 cm^–1^ are attributed to the deformation and elongation of the –NH functional groups. Spectral peaks at 1410 cm^–1^ correspond to the O–H splitting vibrations. Moreover, the peak at around 1025 cm^–1^ originates from the stretching vibration of the C–O–C bonds in the polysaccharide structure.^25^ Comparable peaks were detected in the MPDA/CS and EGCG@MPDA/CS hydrogels, and the inclusion of MPDA NPs and EGCG@MPDA NPs did not lead to the emergence of any new distinctive peaks. Overall, the chemical structure of MPDA/CS and EGCG@MPDA/CS hydrogels remained mostly unchanged, indicating that there was no chemical cross-linking between MPDA NPs/EGCG@MPDA NPs and the CS hydrogel. Physical interactions between the CS hydrogel and MPDA NPs/EGCG@MPDA NPs, including possible hydrogen bonding, may account for the slight changes in the peak position and intensity.^24^ Moreover, the mechanical characteristics of the hydrogels were assessed, as depicted in Figure S4B. Comparable compression moduli were observed among the hydrogels, with the EGCG@MPDA/CS hydrogel having a compression modulus of 11.32 ± 1.525 kPa. The mending effectiveness of hydrogels might, moreover, be influenced by their mechanical characteristics. Rather, pliable hydrogels can enhance the transfer of mechanical impulses and the synchronization of contractions in myocardial tissue, therefore facilitating the restoration of heart function.^26^ The process of adhesion is of utmost importance in cementing the hydrogel to the adjacent cardiac tissue following the injection. The adhesive properties of the hydrogels were analyzed, and the adhesion strengths of the hydrogels were similar (Figure S4C). All hydrogels exhibited the capacity to attach to cardiac tissue, with the EGCG@MPDA/CS hydrogel reaching a peak adhesion strength of 7.067 ± 0.515 kPa.

### 2.3 Evaluation of EGCG Cumulative Release and Degradation of the Hydrogel *In Vitro*

Next, we examined the EGCG release from the EGCG@MPDA/CS hydrogel *in vitro*. In EGCG@MPDA NPs, since no chemical bonds are formed between EGCG and MPDA NPs, EGCG can be gradually released through diffusion. Additionally, the massive generation of ROS during the first phase of MI can disrupt the chemical bonds and structure of PDA, accelerating drug release.^27^ As shown in Figure 2E, the EGCG@MPDA/CS hydrogel achieved 22.25 ± 2.82% EGCG release on day 1, and the cumulative release increased to 40.44 ± 2.60% by day 3. Over time, the cumulative release of EGCG increased, and by day 14, it was at 84.38 ± 1.93%. Over time, the rate of EGCG release reduced progressively. These results suggest that the EGCG@MPDA/CS hydrogel can be used for *in vivo* investigations on ROS scavenging and antiapoptotic therapy following MI since it not only permits rapid release in the early stages but also achieves sustained release over time *in vitro*. Furthermore, we evaluated the hydrogel’s biodegradability (Figure S5). The findings suggested that the EGCG@MPDA/CS hydrogel demonstrated favorable degradability as its weight remained constant at 38.46 ± 3.42% of the original weight after 14 days.

### 2.4 Antioxidant Capacity Detection

Using the 2, 2′-azino-bis (3-ethylbenzothiazoline-6-sulfonic acid) (ABTS) radical-scavenging assay, hydroxyl radical (•OH)-scavenging assay, and 1,1-diphenyl-2-picrylhydrazyl (DPPH) radical-scavenging assay, we assessed the antioxidant activity of the EGCG@MPDA/CS hydrogel in removing ROS in MI heart tissue with high oxidative stress. The CS hydrogel, MPDA/CS hydrogel, and EGCG@MPDA/CS hydrogel all exhibited antioxidant capacity (Figure 2F–H). Figure 2I shows the reaction of the DPPH reagent with the CS hydrogel, MPDA/CS hydrogel, and EGCG@MPDA/CS hydrogel. After a 30 min incubation period without exposure to light, the DPPH solution changed color from a saturated purple shade to yellow upon contact with the EGCG@MPDA/CS hydrogel. This observation emphasizes the remarkable capacity of the EGCG@MPDA/CS hydrogel to scavenge DPPH radicals. The antioxidant characteristics of chitosan hydrogels are attributed to the existence of uncombined amino and hydroxyl groups in chitosan molecules. These groups have the ability to interact with radicals and exhibit antioxidant activity.^28^ Significantly higher radical-scavenging activity was observed in the EGCG@MPDA/CS hydrogel compared to both the CS hydrogel and the MPDA/CS hydrogel (*P* < 0.05). This suggests that incorporating EGCG@MPDA NPs can elevate the antioxidant activity of the CS hydrogel beyond that of pure MPDA NPs. The abundant antioxidant groups brought by EGCG, such as phenolic hydroxyl groups, are the primary factor for the enhancement of antioxidant capacity.^29^ In the MI region, the EGCG@MPDA/CS hydrogel demonstrates remarkable antioxidant properties that enhance the removal of detrimental ROS and reorganize the MI microenvironment.

### 2.5 Protective Effects of the EGCG@MPDA/CS Hydrogel on H9C2 Cells and Human Umbilical Vein Endothelial Cells (HUVECs) in the ROS Microenvironment

**Figure 3:**
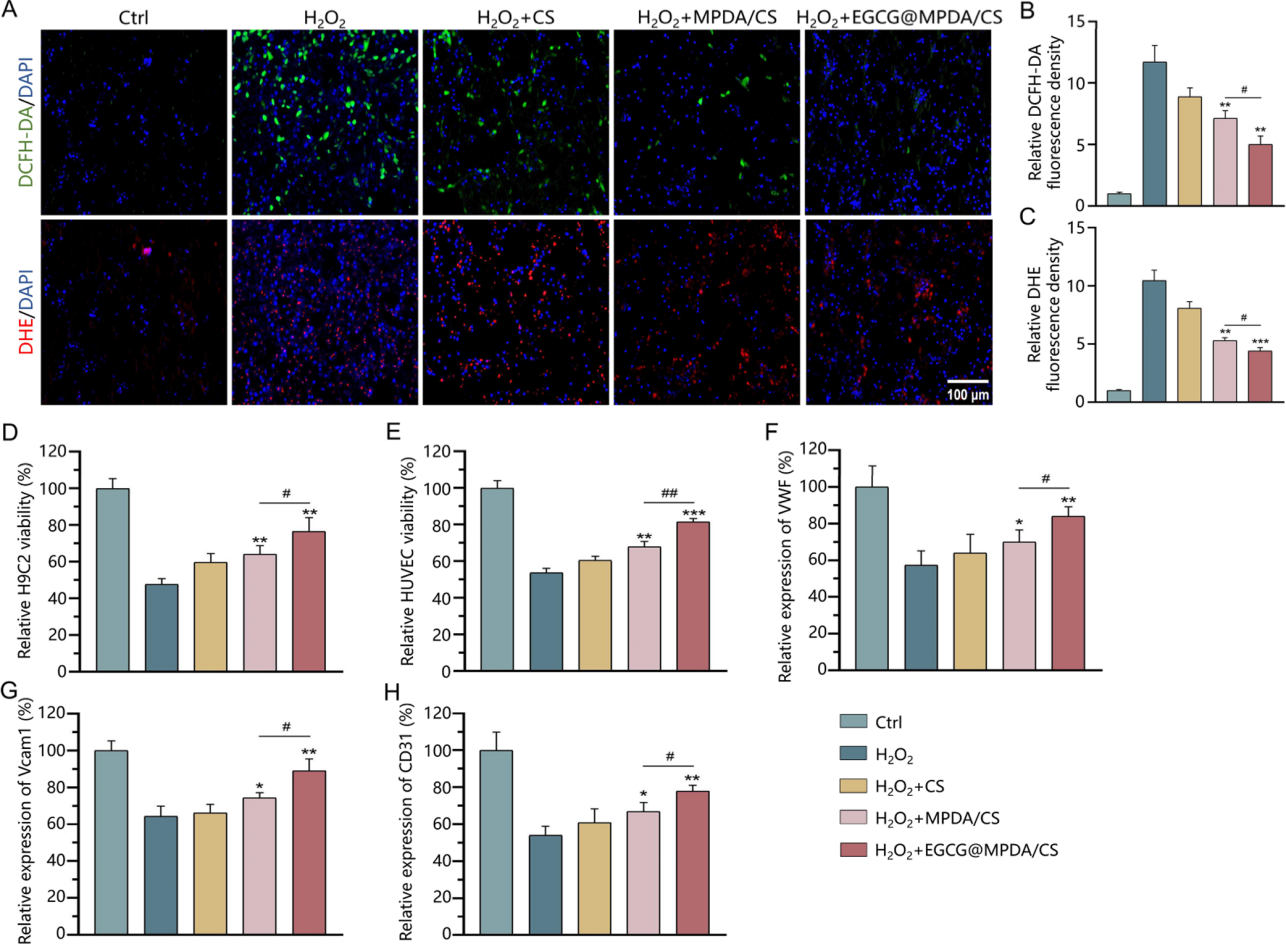
Effect of hydrogels on cells in the ROS microenvironment. (A) Representative fluorescent images of intracellular ROS in each group of cells. Quantification analysis of DCFH-DA (B) and DHE (C) (*n* = 3 for each group). The impact of hydrogels on the viability of H9C2 cells (D) and HUVECs (E) in the ROS microenvironment (*n* = 6 for each group). Expression of genes VWF (F), Vcam1 (G), and CD31 (H) in the ROS microenvironment treated with hydrogels was assessed using qRT-PCR (*n* = 3 for each group). (Results are presented as the mean ± SD.*P < 0.05, **P < 0.01, ***P < 0.001 vs H_2_O_2_ group; ^#^*P* < 0.05, ^##^*P* < 0.01 vs H_2_O_2_ + MPDA/CS group).

We assessed the ability of the EGCG@MPDA/CS hydrogel to remove ROS from H9C2 cells in the ROS microenvironment by measuring intracellular ROS levels using 2′,7′-dichlorofluorescin diacetate (DCFH-DA) and dihydroethidium (DHE) stain assays. Figure 3A illustrates that after 2 days of growth, H9C2 cells in the H_2_O_2_ group showed green fluorescence from DCFH-DA and pronounced red fluorescence from DHE (accompanied by blue fluorescence from DAPI), signifying an elevated level of ROS relative to the Ctrl group. By comparison to the H_2_O_2_ group and H_2_O_2_ + MPDA/CS group, the fluorescence intensity of both DCFH-DA and DHE in the EGCG@MPDA/CS hydrogel-treated group was significantly reduced, as quantitatively analyzed by fluorescence density (Figure 3B,C), indicating that the EGCG@MPDA/CS hydrogel significantly decreased ROS levels in H9C2 cells. Further evaluation of H9C2 viability in the ROS microenvironment (Figure 3D) demonstrated a significant decrease due to oxidative stress damage. The EGCG@MPDA/CS hydrogel exhibited more pronounced enhancement in the survival of H9C2 cells in the condition of an oxidative stress microenvironment compared to the H_2_O_2_ group and H_2_O_2_ + MPDA/CS group. Angiogenic activity is essential for heart function during MI.^30^ Initial evaluation of HUVECs survival in the ROS microenvironment was conducted using the CCK-8 test. Due to oxidative stress damage, significant inhibition of the viability of HUVECs was observed, while the EGCG@MPDA/CS hydrogel partially enhanced endothelial cell viability (Figure 3E). Moreover, the abundance of angiogenesis-related genes, including vascular cell adhesion molecule 1 (VCAM1), von willebrand factor (VWF), and CD31, was evaluated by the use of quantitative real-time polymerase chain reaction (qRT-PCR). As illustrated in Figure 3F, in comparison to the H_2_O_2_ group and H_2_O_2_ + MPDA/CS group, EGCG@MPDA/CS hydrogel treatment significantly upregulated the expression of VWF, VCAM1, and CD31. These results suggest that the EGCG@MPDA/CS hydrogel improved the survival ability of HUVECs, thereby promoting angiogenesis in the MI area.

### 2.6 Inhibition of Apoptosis in H9C2 Cells and Stimulation of the Nrf2/HO-1 Pathway by the EGCG@MPDA/CS Hydrogel in the ROS Microenvironment

**Figure 4:**
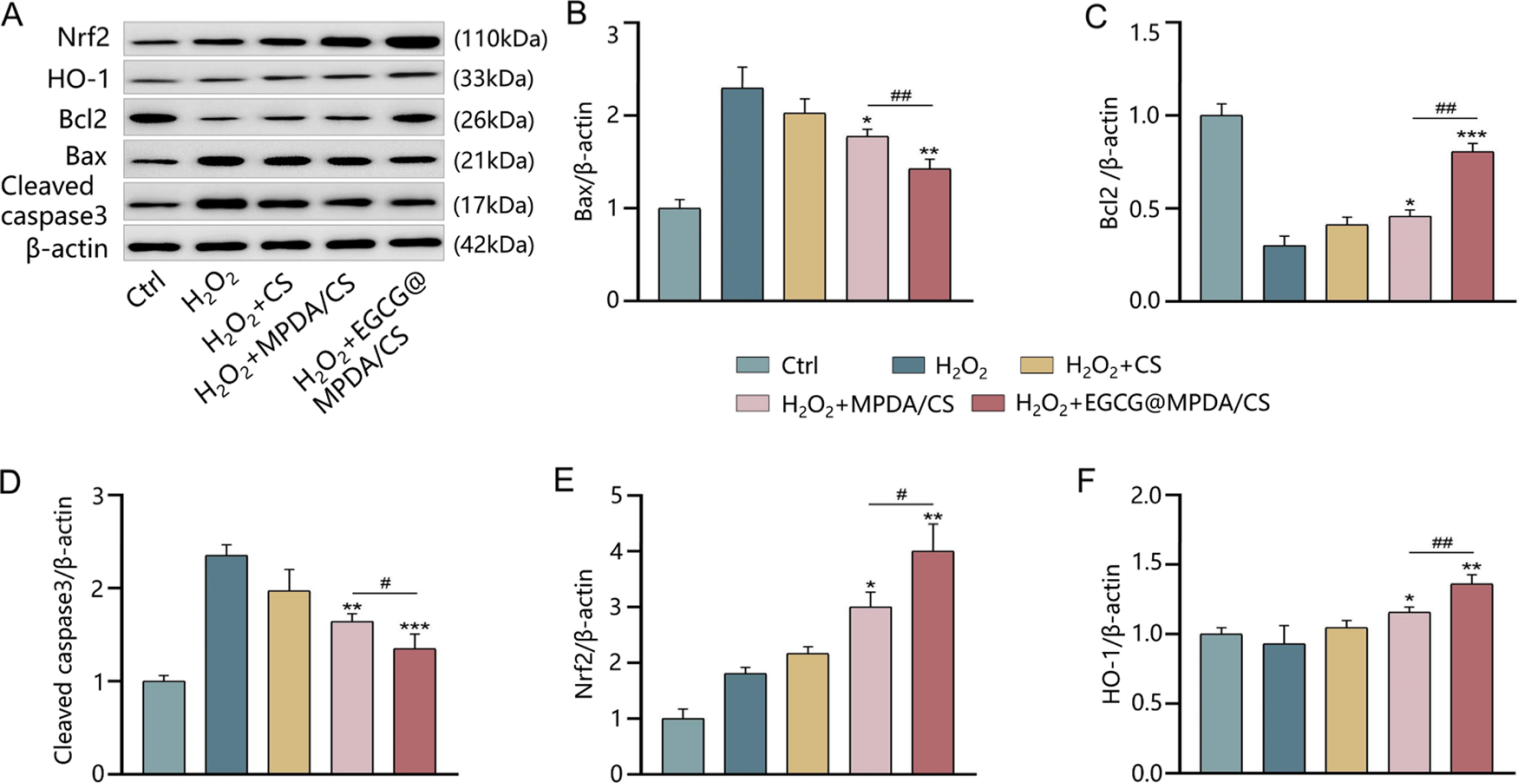
EGCG@MPDA/CS hydrogel inhibits the expression of apoptotic proteins and activates the Nrf2/HO-1 pathway. (A) Representative western blotting of Nrf2, HO-1, Bcl2, Bax, cleaved caspase3, and β-actin. Quantification analysis of Bax (B), Bcl2 (C), cleaved caspase3 (D), Nrf2 (E), and HO-1 (F). (Results are presented as the mean ± SD; *n* = 3 for each group. *P < 0.05, **P < 0.01, ***P < 0.001 vs H_2_O_2_ group; ^#^*P* < 0.05, ^##^*P* < 0.01 vs H_2_O_2_ + MPDA/CS group).

MI typically results in extensive apoptosis of cardiomyocytes.^31^ An inhibitory effect of the EGCG@MPDA/CS hydrogel on apoptosis of H9C2 cells in the ROS environment was evaluated using western blot analysis to examine protein expression associated with the apoptotic pathway. The results depicted in Figure 4A–D demonstrate that the expression of B-cell lymphoma 2 (Bcl2) was dramatically increased in the EGCG@MPDA/CS hydrogel group in comparison to both the H_2_O_2_ group and H_2_O_2_ + MPDA/CS group. Conversely, the levels of Bcl-2-associated X protein (Bax) and cleaved caspase3 were considerably decreased. Bax, Bcl2, and Cleaved caspase3 are all associated with cell apoptosis, where Bcl2 is an anti-apoptotic factor, while Bax and cleaved caspase3 are pro-apoptotic molecules. Therefore, the EGCG@MPDA/CS hydrogel exhibits an inhibitory impact on apoptosis in H9C2 cells subjected to oxidative stress conditions. Stimulation of the Nrf2/HO-1 pathway is essential for the healing of MI as it is directly associated with inhibiting cardiomyocyte apoptosis and protecting cardiomyocytes from oxidative stress.^32^ Hence, we investigated the impact of the EGCG@MPDA/CS hydrogel on the Nrf2/HO-1 pathway (Figure 4E,F). Our findings indicate that treatment with the EGCG@MPDA/CS hydrogel successfully stimulated the Nrf2/HO-1 pathway as compared to the MPDA/CS hydrogel. To validate the participation of the Nrf2/HO-1 pathway in the anti-apoptotic behavior of the EGCG@MPDA/CS hydrogel, we exposed the H_2_O_2_ + EGCG@MPDA/CS group to the Nrf2-specific inhibitor ML385. Following the inhibition of the Nrf2/HO-1 pathway, a significant decrease in the expression of the anti-apoptotic protein Bcl2 was seen, accompanied by a notable increase in the levels of the pro-apoptotic markers Bax and cleaved caspase3 (Figure S6). These results suggest a partial reversal of the anti-apoptotic effect of the EGCG@MPDA/CS hydrogel. These results validate that the EGCG@MPDA/CS hydrogel can stimulate the Nrf2/HO-1 pathway, hence exhibiting anti-apoptotic properties.

### 2.7 *In Vivo* ROS Clearance and Anti-apoptotic and Anti-inflammatory Effects of the EGCG@MPDA/CS Hydrogel

**Figure 5:**
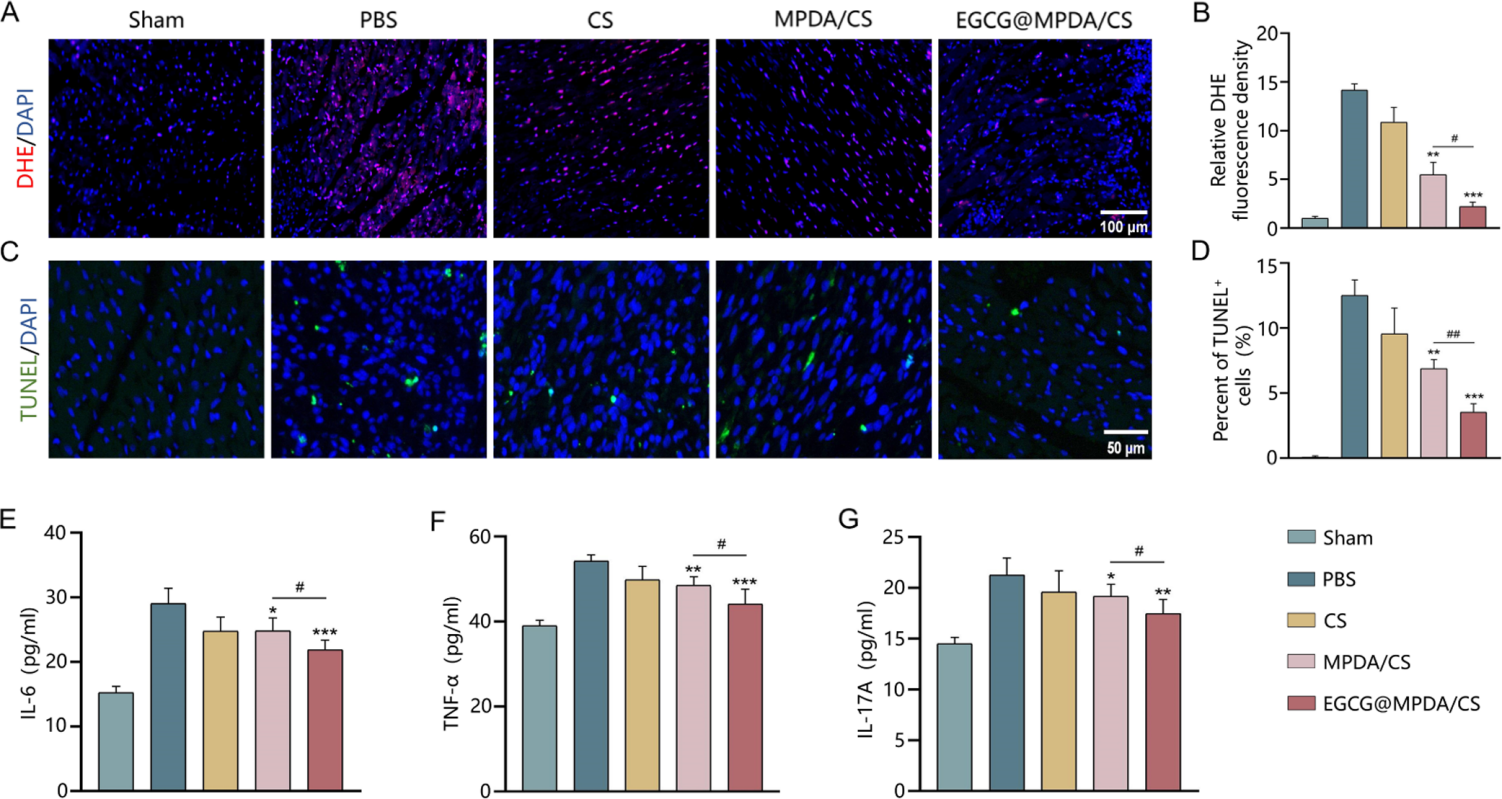
Levels of ROS, apoptosis, and inflammatory factors in five groups of rats. (A) Representative fluorescent images of intracellular ROS in rats from five groups 3 days after MI. (B) Quantitative analysis of DHE (*n* = 3 for each group). (C) Representative TUNEL staining images of 3 days after MI. (D) Quantitative analysis of TUNEL staining (*n* = 3 for each group). Quantitative analysis of IL-6 (E) and TNF-α (F) levels 3 days following MI (*n* = 6 for each group). Quantitative analysis of IL-17A (G) levels 7 days after MI (*n* = 6 for each group). (Results are presented as the mean ± SD. *P < 0.05, **P < 0.01, ***P < 0.001 vs PBS group; ^#^*P* < 0.05, ^##^*P* < 0.01 vs MPDA/CS group).

We further investigated the effect of the EGCG@MPDA/CS hydrogel on *in vivo* ROS clearance. The schematic diagram of model construction and drug delivery is shown in Figure S7. On day 3 after MI treatment, we used DHE staining to reveal the amounts of ROS in the tissues. As shown in Figure 5A,B, the PBS group showed intense red fluorescence, indicating high ROS levels. Comparisons between the PBS group and the MPDA/CS group showed that the fluorescence level in the EGCG@MPDA/CS group was significantly reduced, suggesting a significant reduction in ROS levels *in vivo* by the EGCG@MPDA/CS hydrogel. Cellular apoptosis occurs during the early stages of an infarction, lasting from hours to a week, and directly affects the later remodeling of the heart and its function.^33^ Anti-apoptotic signaling pathways can enhance the survival of cardiomyocytes.^34^ To identify apoptotic cells in the border zone of the infarcted area, we used terminal deoxynucleotidyl transferase-mediated dUTP nick end labeling (TUNEL) on the third day following MI. In the border zone of the infarcted area, the group treated with the EGCG@MPDA/CS hydrogel exhibited the lowest occurrence of cardiomyocyte apoptosis among all the groups with MI (Figure 5C,D). Additionally, the protective effect of the EGCG@MPDA/CS hydrogel against cell apoptosis was stronger than that of the MPDA/CS hydrogel. Thus, the findings indicate that the EGCG@MPDA/CS hydrogel can efficiently lower the rate of cell apoptosis in ischemic myocardium, potentially supporting the reduction of ventricular remodeling and enhancement of cardiac function following MI.

The inflammatory response is a crucial healing step that occurs after MI in addition to cell death. Extended inflammation following MI can result in increased fibrosis, accelerated matrix breakdown, and widespread cardiomyocyte loss.^35^ Thus, a possible therapeutic strategy after MI is to reduce the inflammatory response.^36^ Tumor necrosis factor-α (TNF-α) and interleukin-6 (IL-6) are believed to be significant early inflammatory agents in myocardial ischemia injury, reaching their peak in the first 3 to 4 days after the injury.^37^ Interleukin-17A (IL-17A) can induce cardiomyocyte apoptosis and cardiac fibrosis, peaking at day 7 after MI.^38^ Therefore, in this study, we assessed the levels of TNF-α and IL-6 on the third day after MI and IL-17A on the seventh day after MI using an enzyme-linked immunosorbent assay (ELISA). The findings indicate that rats administered with the EGCG@MPDA/CS hydrogel exhibited significantly reduced levels of expression of these three inflammatory factors in comparison to the groups treated with PBS and the MPDA/CS hydrogel (Figure 5E–G). This indicates that the EGCG@MPDA/CS hydrogel therapy successfully suppresses these inflammatory factors in MI. These results demonstrate that the EGCG@MPDA/CS hydrogel treatment effectively suppresses the elevation of inflammatory factors in MI.

### 2.8 Improvement of Cardiac Function by the EGCG@MPDA/CS Hydrogel

**Figure 6:**
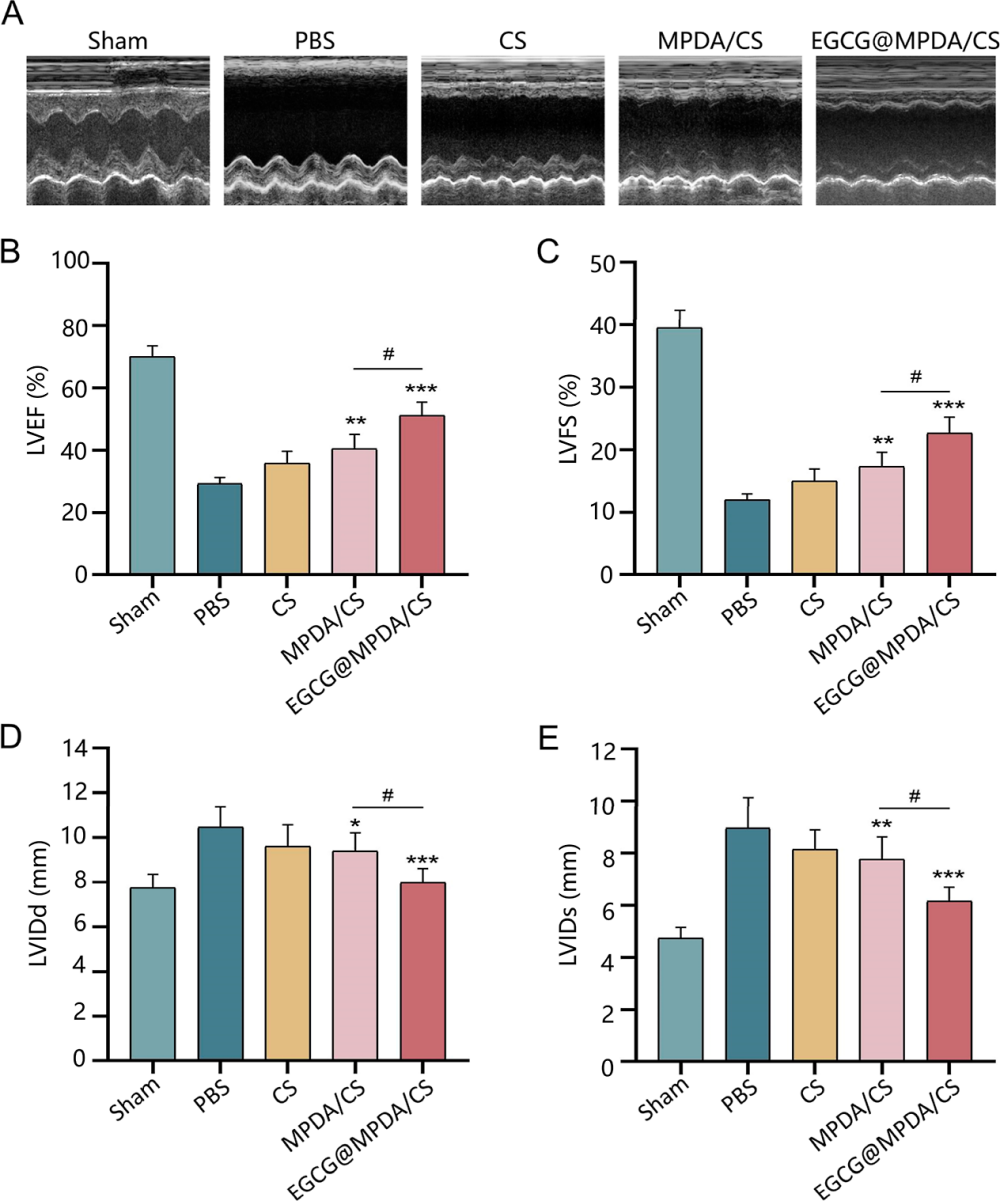
Echocardiographic measurements of cardiac function in five groups of rats 28 days after MI (*n* = 6 for each group). (A) Representative echocardiogram images from the five rat groups. (B–E) Quantitative analysis of the LVEF value (B), LVFS value (C), LVIDd (D), and LVIDs (E) in the five groups of rats. (Results are presented as the mean ± SD. *P < 0.05, **P < 0.01, ***P < 0.001 vs PBS group; ^#^*P* < 0.05 vs MPDA/CS group).

Next, we tested whether the EGCG@MPDA/CS hydrogel could exert reparative effects on the hearts of rats with MI. Echocardiography, as a noninvasive modality, is the main approach for assessing cardiac function. The therapeutic benefits of heart repair in rats 28 days after MI were evaluated using echocardiography (Figure 6A). Following MI, the PBS group of rats exhibited significant cardiac dysfunction, as evidenced by a marked decrease in left ventricular ejection fraction (LVEF) and left ventricular fraction shortening (LVFS) values. The efficacy of the pure CS hydrogel in cardiac repair was shown to be restricted, leading to an elevation in LVEF and a reduction in left ventricular internal dimension in systole (LVIDs). The CS hydrogel improved LVEF by approximately 6%, which is consistent with previous reports.^39^ Notably, in the EGCG@MPDA/CS hydrogel group, LVEF and LVFS significantly increased, from 29.31 ± 0.78% to 51.25 ± 1.73% and from 12.02 ± 0.37% to 22.66 ± 1.06%, respectively (Figure 6B,C), while LVIDs and left ventricular internal dimension in diastole (LVIDd) significantly decreased (Figure 6D,E), indicating a marked recovery of cardiac function. The results demonstrated that the EGCG@MPDA/CS hydrogel increased LVEF by approximately 20%, confirming its superior myocardial repair performance. The system demonstrated an enhancement in performance of around 10% to 15% compared to other systems targeting the MI microenvironment.^40,41^ A more extensive investigation is required to examine the enduring impacts of the EGCG@MPDA/CS hydrogel.

### 2.9 Promotion of Angiogenesis and Attenuation of Ventricular Remodeling by the EGCG@MPDA/CS Hydrogel

**Figure 7:**
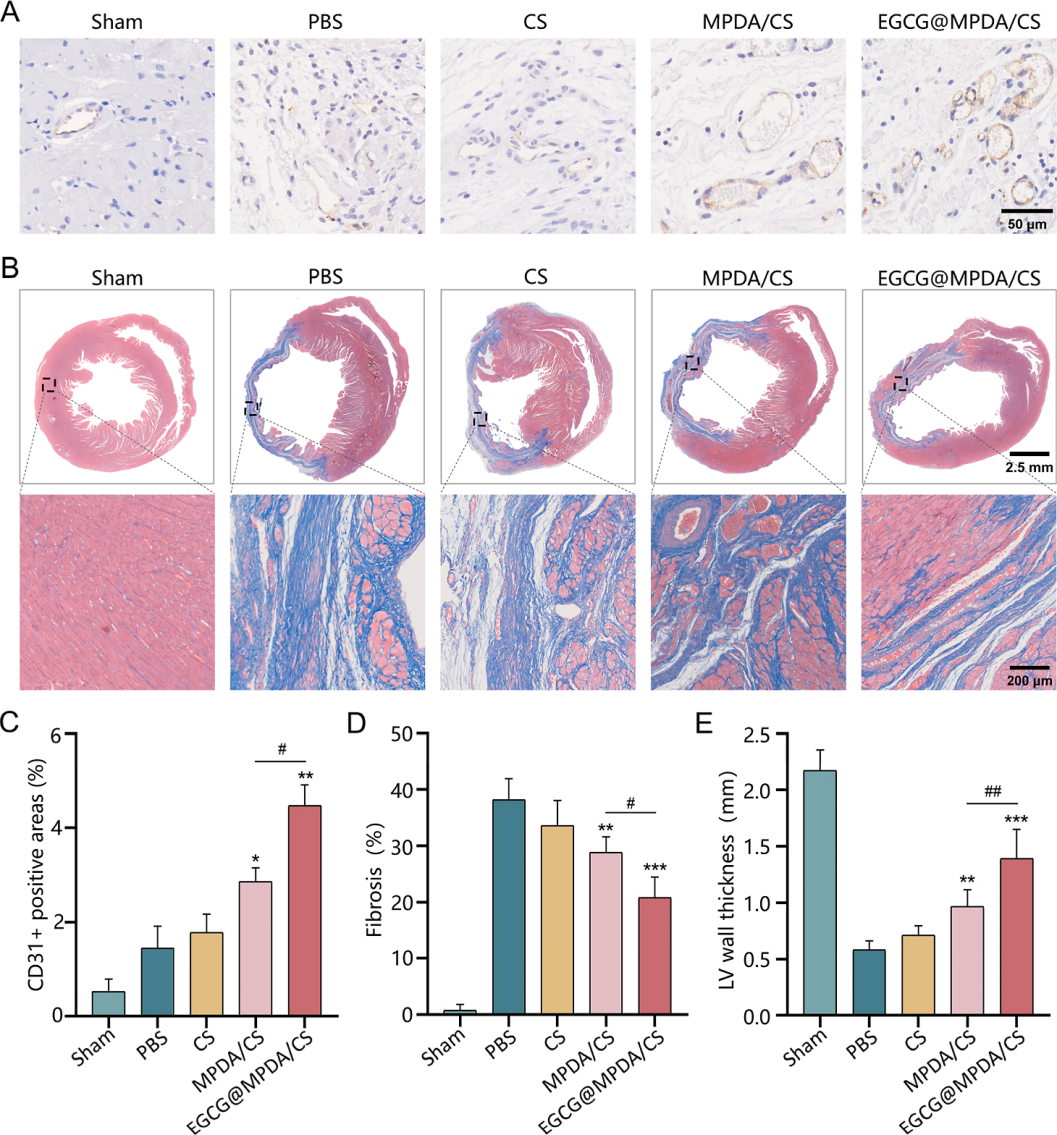
Evaluation of cardiac remodeling and angiogenesis in five groups of rats 28 days after myocardial infarction (*n* = 6 for each group). (A) Representative images of CD31 immunohistochemical staining in cardiac tissue samples from the five rat groups. (B) Representative images of Masson’s trichrome staining in cardiac tissue samples of the five groups of rats. (C) Quantitative evaluation of the five groups of rats’ heart sections’ CD31 area percentage. Quantitative analysis of fibrosis (D) and LV wall thickness (E) in the five rat groups. (Results are presented as the mean ± SD. *P < 0.05, **P < 0.01, ***P < 0.001 vs PBS group; ^#^*P* < 0.05 vs MPDA/CS group).

After MI, coronary revascularization is crucial for restoring cardiac function,^42^ and increased angiogenesis can promote oxygen and nutrient supply to the infarct area. Previous research has indicated that EGCG possesses the capacity to protect endothelial cells from harm induced by the condition of oxidative stress and suppress inflammation in these cells, therefore enhancing their survival.^43−45^ Therefore, the EGCG@MPDA/CS hydrogel loaded with EGCG is expected to have a strong angiogenic effect. We assessed angiogenesis in rat myocardium 28 days post MI using endothelial cell CD31 staining. The CD31 staining area was measured to be 1.45 ± 0.26% in the PBS group and 2.86 ± 0.17% in the MPDA/CS hydrogel group, as shown in Figure 7A,C. Remarkably, this value significantly rose to 4.47 ± 0.25% in the EGCG@MPDA/CS hydrogel group, indicating the most significant angiogenesis promotion by the EGCG@MPDA/CS hydrogel, consistent with the *in vitro* experimental results.

Ventricular remodeling is a key factor in cardiac dysfunction after MI, frequently characterized by myocardial fibrosis, decreased thickness of the damaged wall, and the development of scar tissue.^46^ Thus, the evaluation of remodeling after an infarction needed the consideration of two crucial factors: myocardial fibrosis and left ventricular wall thinning. An evaluation of cardiac remodeling in rats 28 days post MI was conducted using Masson’s trichrome staining. Figure 7B shows that blue staining revealed notable fibrosis in the myocardium of infarcted rats in the PBS group, characterized by a decreased thickness of LV walls in comparison to the sham group. Consistent with the echocardiographic results, treatment with the EGCG@MPDA/CS hydrogel significantly reduced collagen deposition and minimized the weakening of the LV wall. Following treatment with the EGCG@MPDA/CS hydrogel, the fibrosis ratio decreased from 38.17 ± 1.546% to 20.8 ± 1.494%, and the thickness of the LV wall increased from 0.58 ± 0.03 to 1.39 ± 1.11 mm in comparison with the PBS group (Figure 7D,E). However, the effectiveness of the MPDA/CS hydrogel in reducing ventricular fibrosis and remodeling was notably inferior compared to that of the EGCG@MPDA/CS hydrogel. The results firmly confirm that the EGCG@MPDA/CS hydrogel has the most effective therapeutic effects on cardiac structure and function after MI. Furthermore, the leukocyte levels in the EGCG@MPDA/CS hydrogel group did not exhibit any notable disparities when compared to the PBS group at 1, 3, and 7 days after MI treatment (Figure S8), indicating that the injection of the EGCG@MPDA/CS hydrogel did not induce additional inflammatory responses. Hematoxylin and eosin (H&E) staining of vital organs (heart, spleen, liver, kidney, and lung) after 28 days revealed no indications of inflammation or pathological alterations occurring in the organs (Figure S9). It was determined that all of the hydrogels exhibited favorable safety profiles.

## 3 Conclusions

We developed an EGCG@MPDA/CS hydrogel that is biocompatible and effectively decreases ventricular remodeling to enhance cardiac recovery following MI. This hydrogel consisted of the CS hydrogel and EGCG@MPDA NPs, with EGCG@MPDA NPs cross-linking with the CS hydrogel via intermolecular hydrogen bonds and distributed uniformly across the CS hydrogel matrix. *In vitro* experiments demonstrate that the EGCG@MPDA/CS hydrogel exhibited excellent ROS-scavenging activity, significantly reduced intracellular ROS levels in H9C2 cells under oxidative stress conditions, and inhibited apoptosis of H9C2 cells. Administration of the EGCG@MPDA/CS hydrogel into the damaged heart muscle of rats following MI successfully suppressed the production of inflammatory factors, therefore reducing the apoptosis of cardiomyocytes. Therapy with the EGCG@MPDA/CS hydrogel for 28 days in rats with MI greatly improved the recovery of cardiac function and strongly decreased myocardium fibrosis and remodeling in the infarcted region. The results indicate that the EGCG@MPDA/CS hydrogel developed in this research offers a valuable approach to enhance cardiac repair following MI and holds promise for clinical translation. Moreover, this anti-inflammatory and anti-apoptotic hydrogel design strategy may offer novel perspectives and approaches for addressing inflammatory conditions.

## 4 Materials and Methods

### 4.1 Synthesis and Analysis of MPDA NPs and EGCG@MPDA NPs

The preparation of MPDA NPs was carried out following established methods.^19,47^ In brief, Pluronic F127 (1 g) (Macklin, China) and TMB (1 g) (Macklin, China) were added to a 100 mL mixture of ethanol and water in a 1:1 v/v ratio. For 30 min, the reaction mixture was stirred by magnetic stirring to generate a white emulsion. Next, a 3.75 mL ammonia solution (Aladdin, China) and 1.5 g of dopamine hydrochloride (Macklin, China) were introduced, and the reaction was thereafter carried on at ambient temperature for 2 h. Centrifugation of the suspension was performed at 13,000 rpm for 10 min. The resulting precipitate was then gathered and rinsed twice with a water/ethanol (1:1, v/v) mixture. Following a 24 h vacuum-drying process, the precipitate was transformed into black MPDA NPs powder.

EGCG was purchased from Macklin (China). Following the dissolution of 50 mg of EGCG in 100 mL of water, 100 mg of MPDA NPs was introduced. The solution was stirred at ambient temperature overnight and subjected to centrifugation at 13,000 rpm for 10 min, and the resultant solid was gathered and dried under vacuum to produce EGCG@MPDA NPs.

A UV–vis spectrophotometer (UV-2000, UNICO, USA) calibrated against the standard curve for the drug loading estimate was used to quantify the concentration of EGCG in the supernatant. We analyzed the structures of MPDA NPs and EGCG@MPDA NPs using TEM (JEM-2100F, Jeol, Japan). The zeta potential and particle size of MPDA NPs and EGCG@MPDA NPs were measured using a nanoparticle size analyzer (NanoBrook 90Plus PALS, Brookhaven, USA). FTIR (Nicolet iS20, Thermo Scientific, USA) was employed to confirm the EGCG loading in the EGCG@MPDA NPs.

### 4.2 Preparation and Characterization of Hydrogels

The temperature-responsive CS hydrogel was synthesized following the methods described in the literature.^48^ In brief, a 0.1 M acetic acid solution (Macklin, China) was prepared, and 2% chitosan (w/v, in 0.1 M acetic acid solution) (Macklin, Product No: C804728) was sterilized by conventional liquid autoclaving. Subsequently, a solution containing 11.5% β-GP (w/v) was formulated in distilled water sourced from Macklin, China, by using filtration and sterilization techniques. The formation of the CS hydrogel involved the combination of 8 mL of chitosan with 2 mL of β-GP solution, followed by incubation at 37 °C. The MPDA/CS hydrogel and EGCG@MPDA/CS hydrogel were prepared similarly using the same method. Different amounts of MPDA NPs and EGCG@MPDA NPs were introduced into a mixed solution of chitosan and hydroxyethyl cellulose (2.5 mL, 2.5% (w/v) in PBS) (Macklin, China). The concentrations of MPDA NPs and EGCG@MPDA NPs were 0, 0.2, 0.5, 1.0, 2.0, and 3.0 mg/mL, respectively. Then, the β-GP solution was added to the mixture solution. CS hydrogels and hydrogels with different concentrations of MPDA/CS and EGCG@MPDA/CS could be formed within 5 min.

Using a sputter coater, a layer of gold was applied to the freeze-dried hydrogels before observation. Next, we investigated the microstructure of the samples using SEM (JSM-6380, JEOL, Japan) at an accelerating voltage ranging from 10 to 20 kV. The rheological characteristics of different hydrogels were evaluated at 37 °C using the HAAKE Rehostress RS6000 rheometer. FTIR was employed to record the spectra of the CS, MPDA/CS, and EGCG@MPDA/CS hydrogels. An optical resolution of 4 cm^–1^ was attained over the whole spectral range spanning from 4000 to 400 cm^–1^. The stability of the hydrogels was tested based on the method outlined in prior research.^49^ Records of the *G*′ and *G*″ curves were obtained by performing a frequency sweep test covering a range of 0.1–10 Hz while subjecting the material to a load of 20 Pa. The mechanical characteristics of the hydrogels were evaluated by a compression test using the experimental procedure outlined in the literature.^50^ Compression testing at 37 °C was conducted on hydrogel samples that were formed into cylinders measuring 10 mm in diameter and 5 mm in height. The experiments were carried out using a universal testing machine (Instron MTS) integrated with a 50 N load cell, running at a rate of 1 mm/min at 80% strain. All tests were repeated 5 times. Additionally, the hydrogels’ adhesion capacity to fresh porcine skin was evaluated. 10 mm × 30 mm strips of porcine skin were cut and then submerged and stored in a PBS solution. Following the drying of the porcine skin’s surface, hydrogel solution samples were applied onto it, with an additional piece of porcine skin coated with the same hydrogel sample positioned on top. The porcine skin, coupled with the hydrogel layer, was subjected to a 10 min incubation at 37 °C. Before performing the adhesion assessment, the porcine skin in question was left at ambient temperature for a duration of 2 h. The bonding area was then measured to be 10 × 10 mm. Using a material testing apparatus (MTS Criterion 43, MTS Systems), the adhesion performance of the matrix was assessed through lap shear testing at a rate of 2 mm per minute. All experiments were conducted in triplicate to ensure the reliability and uniformity of the results.

### 4.3 Cell Culture

Cultivated in 25 cm^2^ cell culture flasks, H9C2 cells were obtained from Servicebio (China). The cells were transferred to a cell culture incubator and set at 37 °C with 5% CO_2_ in DMEM (Procell, China) containing 1% P/S (penicillin/streptomycin) and 10% fetal bovine serum (FBS) (Procell, China). Once 80% confluency was achieved, the culture medium was changed every 2 days, and cell passaging was performed. The HUVECs were obtained from Oricell in China and grown in endothelial cell medium (ECM) (ScienCell, USA). The ECM included 1% endothelial cell growth substrate and 5% FBS. The cultivation was carried out at 37 °C including 5% CO_2_.

### 4.4 Cell Viability Assay

#### 4.4.1 Relative Cell Viability Assay

Cell viability and proliferation of H9C2 were evaluated by the CCK-8 test. The culture medium of H9C2 cells was supplemented with the EGCG@MPDA/CS hydrogels at concentrations of 0, 0.2, 0.5, 1, 2, and 3 mg/mL. The culture medium was then withdrawn after 24 and 48 h. Subsequent to aspiration of the cell culture medium, a 10% CCK-8 solution (Biosharp, China) was added. The cells were thereafter cultured for an additional 4 h. An optical microplate reader (SpectraMax M5, Molecular Devices, USA) was used to quantitatively measure the absorbance of the supernatant at 450 nm. Viability of the cells was evaluated by comparing them to control cells that were separated and solely treated with a culture medium. After screening the concentration of the EGCG@MPDA/CS hydrogel for use, the H9C2 cells were cultured with a pure CS hydrogel and MPDA/CS and EGCG@MPDA/CS hydrogels at this concentration for up to 7 days; cellular viability was assessed at 1, 3, and 7 days, respectively. The viability of H9C2 and HUVECs treated with a hydrogel under oxidative stress conditions was assessed using the same method. The calculation method for relative cell viability is as follows
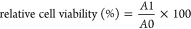


In this context, *A*0 represents the absorbance of the control group, while *A*1 represents the absorbance of the treatment group.

#### 4.4.2 Live/Dead Cell Staining

Staining of live/dead cells was conducted on H9C2 cells that were grown with CS, MPDA/CS, and EGCG@MPDA/CS hydrogels for 7 days. Following thorough washing with PBS, the cells were exposed to a staining solution comprising calcein-AM and propidium iodide (Beyotime, China) at ambient temperature for 15 min. Fluorescence images were acquired by using a fluorescence microscope (Olympus, Japan).

#### 4.4.3 Hemolysis Assessment

The hemolysis assessment was conducted following the methods described in previous literature.^51^ Hydrogel samples were immersed in test tubes filled with a 10 mL solution of sodium chloride. There were separate test tubes assigned to the control groups, each filled with 10 mL of Triton solution (positive control) and PBS (negative control). Proceeding, the tubes were submerged in a water bath maintained at a temperature of 37 °C for a period of 30 min. Subsequently, rabbit blood that had been diluted was delicately blended into each tube. The tubes were subjected to a 60 min continuous incubation at 37 °C, followed by centrifugation at 800*g* for 5 min. A 545 nm wavelength optical microplate reader was used to quantify the absorbance of the supernatant. Three replicates of each measurement were performed to guarantee the precision. The hemolysis rate of the hydrogels was determined by computation using the provided formula
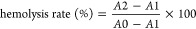


The absorbance of the positive control group is denoted by *A*0 in the formula, the negative control group’s absorbance is denoted by *A*1, and the absorbance of the hydrogel group is denoted by *A*2.

### 4.5 Investigation of EGCG Release from the EGCG@MPDA/CS Hydrogel *In Vitro*

The release properties of EGCG were investigated by preparing 1 mL of the EGCG@MPDA/CS hydrogel in centrifuge tubes. Subsequently, 10 mL of PBS solution was introduced into the tubes, which were then positioned in an incubator set at 37 °C and continuously agitated at a speed of 100 rpm. Specified time intervals were used to extract 100 μL of solution from each tube and combine it with 100 μL of fresh PBS. The samples underwent centrifugation at a force of 14,800 *g* for 10 min. The EGCG concentration was established by quantifying the absorbance at a spectrum wavelength of 276 nm using a spectrophotometer. Each sample was tested in triplicate.

### 4.6 Assessment of Hydrogel Degradation *In Vitro*

The hydrogels were subjected to an *in vitro* degradation experiment using the techniques established in prior research.^51^ The hydrogel samples underwent freeze-drying, followed by weighing and recording as *A*0. Subsequently, the samples were submerged in a PBS solution and positioned in a temperature-controlled shaker set at 37 °C and 70 rpm. Individual hydrogel samples were removed, freeze-dried, weighed, and labeled as *A*1 at predetermined time intervals of 1, 3, 7, and 14 days. The residual mass ratio of the hydrogel samples in the *in vitro* setting was determined by utilizing the subsequent equation
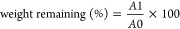


### 4.7 Antioxidant Activity Assay of the EGCG@MPDA/CS Hydrogel

#### 4.7.1 DPPH Radical Scavenging

The antioxidant potential of the hydrogels was assessed by measuring their DPPH radical-scavenging activity. The experimental procedure was carried out with minor adjustments based on the method outlined in the literature.^52^ In brief, a total of 100 μL of CS, MPDA/CS, and EGCG@MPDA/CS hydrogels were combined with 900 μL of a 0.2 mM solution of DPPH ethanol. The mixture was allowed to undergo a reaction without exposure to light at a temperature of 25 °C for a period of 30 min. Furthermore, the absorbance of the solution was quantified at a wavelength of 515 nm by using a spectrophotometer. Groups designated as blank and standard controls were subjected to identical reaction conditions. To determine the DPPH radical-scavenging activity of the samples, the following calculation was used.
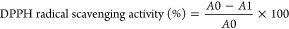


The absorbance of the DPPH standard solution is denoted by *A*0, while the absorbance of the sample solution is encoded by *A*1.

#### 4.7.2 ABTS Radical Scavenging

Evaluation of the ABTS radical-scavenging activity was conducted using a method used in a prior study with minor adjustments.^53^ A 7 mM ABTS solution (Solarbio, Beijing) was made by mixing it with 2.45 mM potassium persulfate (Macklin, China) and then stored in the dark at an ambient temperature for 12 h. Following this, 100 μL of different concentrations of the hydrogel was combined with 900 μL of the ABTS radical solution. In the absence of light, the combination was allowed to perform a reaction at an ambient temperature of 25 °C for a period of 30 min. The absorbance of the combined solution was measured by using an optical microplate reader at a wavelength of 734 nm. An experimental control was created by employing a blank solution devoid of the hydrogel. The method employed to evaluate the ABTS radical-scavenging activity of the samples was as follows
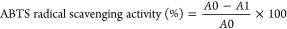


The absorbance of the sample solution is denoted as *A*1, while the absorbance of the ABTS standard solution is denoted as *A*0.

#### 4.7.3 •OH Scavenging

According to previous reports, •OH was generated via the Fenton reaction with slight modifications.^54^ In brief, a solution of FeSO4 (2 mM, 600 μL) (Macklin, China) was mixed with a solution of phenanthroline (360 μg/mL, 500 μL) (Solarbio, Beijing). This mixture was then combined with 300 μL of the prepared CS, MPDA/CS, and EGCG@MPDA/CS hydrogels. For the blank and control groups, deionized water (300 μL) was used instead of the CS, MPDA/CS, and EGCG@MPDA/CS hydrogels. After a 10 min incubation period, the mixture was supplemented with 800 μL of H_2_O_2_ (0.06 wt %) or water (as the control). Furthermore, the measurement of the absorbance of the reaction mixture was conducted at a specific wavelength of 532 nm. The values for the •OH scavenging activity of the samples can be achieved by the following formula
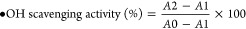


The coordinates *A*0, *A*1, and *A*2 correspond to the absorbance values of the control group, blank group, and experimental group, respectively.

### 4.8 Construction of an Oxidative Stress Cell Model and Intracellular ROS Detection

An oxidative stress cell model was constructed according to the established techniques from literature to simulate the oxidative stress environment after MI.^55^ H9C2 cells and HUVECs were cultured in medium for 24 h. Next, a solution of 200 μM H_2_O_2_ (Macklin, China) was added to the medium, followed by placing the cells in a cell culture incubator and cultivating them for 48 h. Following replacement of the medium, CS, MPDA/CS, and EGCG@MPDA/CS hydrogels were introduced into the medium, and the cells were then cultivated for an extra 48 h. The Nrf2 blocking experiment was conducted by adding ML385 (Macklin, China) and EGCG@MPDA/CS hydrogel simultaneously to the culture medium. H9C2 cells were subsequently exposed to 10 μM DHE (Beyotime, China) or DCFH-DA (Servicebio, China) at 37 °C for 30 min in the absence of light. Subsequently, the cells were subjected to DAPI solvent (Beyotime, China) and allowed to incubate at ambient temperature for 10 min. Imagery was acquired with a fluorescence microscope. The fluorescence intensity of DCFH-DA and DHE was calculated using the IPWIN60 program, which was developed by Media Cybernetics, Inc.

### 4.9 Western Blot Analysis

Protein extraction from H9C2 cells was performed using RIPA buffer (Sigma-Aldrich, USA) containing phosphatase and protease inhibitors in a 9:2:100 ratio, while the cells were stored on ice. Acquired from Solarbio, China, the extracted proteins were quantified by using a BCA protein assay kit. All proteins were separated using a 10% SDS–polyacrylamide gel and then transferred to a PVDF membrane obtained from Millipore, USA. Furthermore, the membrane was hermetically sealed using a 5% (w/v) skim milk solution in TBST for 2 h at ambient temperature. Subsequently, the membrane was left to incubate at 4 °C overnight with the specified primary antibodies: Bax (Abcam, UK, ab32503), Bcl2 (Abcam, UK, ab32124), cleaved caspase3 (Cell Signaling Technology, USA), Nrf2 (Abcam, UK, ab62352), HO-1 (Abcam, UK, ab189491), and β-actin (Servicebio, China, GB15003-100). After undergoing three washes with TBST, the membrane was subsequently subjected to the antirabbit secondary antibody (Beyotime, China, A0208) at ambient temperature for a duration of 4 h. The ECL chemiluminescent reagent (Millipore, USA) was used to visualize the labeled proteins. The grayscale values of the protein bands on the membrane were measured and analyzed utilizing Image J software from NIH, USA.

### 4.10 qRT-PCR Analysis

HUVECs were targeted for total RNA isolation using a total RNA extraction kit from Vazyme, China. Through reverse transcription using the Vazyme cDNA synthesis kit (Vazyme, China), the RNA was transformed into cDNA. The qRT-PCR analysis used β-actin as the internal reference gene utilizing the qPCR SYBR Green Master Mix from Vazyme, China. The primer sequences are given in Table 1.

**Table 1:** 

Gene	Forward (5'-3')	Reverse (5'-3')
VWF	CCTTGACCTCGGACCCTTATG	GATGCCCGTTCACACCACT
VCAM1	GGATAATGTTTGCAGCTTCTCAAG	CATTGTCAGCGTAGATGTGGT
CD31	CCAAGGTGGGATCGTGAGG	TCGGAAGGATAAAACGCGGTC

### 4.11 Creation of the MI Model and Hydrogel Injection

The male SD rats, weighing 200 ± 10 g, were acquired from Sibeifu (Beijing) Biotechnology Co., Ltd. and acclimated for 1 week to ensure good health status. A continuous 12 h light/dark cycle, humidity levels ranging from 50 to 60%, and a temperature of 22 ± 2 °C were maintained to regulate housing conditions. The rats were given standard laboratory chow and access to freely sterile water freely. The MI model was prepared as described previously.^56^ Preoperatively, rats were fasted for 12 h and then anesthetized by administering a 2% pentobarbital sodium intraperitoneal injection (Sigma-Aldrich, USA) at a dosage of 40 mg/kg. Verification of the level of anesthesia was achieved by the absence of a reaction to toe pinching. A small animal laryngoscope was used to expose the trachea, and an endotracheal tube was inserted and connected to a ventilator (Alcbio, China) with parameters set at 80 breaths/min and a 1.5 mL tidal volume for adequate ventilation during surgery. To do a left thoracotomy, the left anterior descending coronary artery (LAD) was tied off immediately above using a 6–0 polypropylene suture. Coronary infarction induction was verified by the presence of local myocardial whitening and ST-segment elevation on an electrocardiogram (ECG). MI model rats were randomly allocated into 5 groups (*n* = 6 per group): sham (no MI, only thoracotomy without LAD ligation), PBS (100 μL), the CS hydrogel (100 μL), the MPDA/CS hydrogel (100 μL), and the EGCG@MPDA/CS hydrogel (100 μL). A 30-gauge needle was used to administer three site injections of hydrogels into the border zone of the infarcted region with each injection delivering 33 μL. Care was taken to stabilize the needle to avoid additional injury to the heart. The chest incision was closed in layers, and the rats recovered on a 37 °C heating pad. Postoperative care included antibiotics and monitoring. The animal experimental techniques in this work were carried out following the guidelines in the Guide for the Care and Use of Laboratory Animals (eighth edition, 2011) by NIH and approved by the IACUC of the General Hospital of the PLA in Beijing, China.

### 4.12 Detection of Apoptosis, Tissue ROS, and Inflammatory Factor Levels after MI

Apoptotic cells in the peri-infarct region were identified by TUNEL on the third day after surgery. The TUNEL staining kit was purchased from Servicebio (China). Sections were counterstained with DAPI to label cell nuclei. Imagery was acquired using an Olympus microscope manufactured in Japan. The TUNEL-positive nuclei or total nuclei were quantitatively analyzed by using the Image J software. To establish the apoptotic index, the number of TUNEL-positive cells was divided by the total number of cells that could be observed in the field of view. The DHE staining reagent was treated with sections from each group for 30 min. Following the washing with PBS, heart slices were treated with DAPI for the purpose of counterstaining and subsequently visualized using a fluorescence microscope. The intensity of diffusive fluorescence of DHE was quantified by using IPWIN60 software. Quantification of inflammatory factor levels was performed using ELISA. Postoperative blood samples were collected from the caudal vein of rats on days 3 and 7. Differential quantification of TNF-α, IL-17A, and IL-6 in each serum sample was performed using appropriate ELISA kits obtained from ABclonal, China.

### 4.13 Assessment of Cardiac Function

Transthoracic echocardiography with a VINNO 6 device equipped with a 6 MHz image transducer was used to evaluate LVEF, LVFS, LVIDs, and LVIDd on the 28th day following MI as part of the postoperative assessment. M-mode echocardiography was performed at the level of the papillary muscle.

### 4.14 Pathological and Immunohistochemical Analysis

Humane euthanasia of rats was performed, and their hearts were collected on the 28th day following a MI surgery. The tissue was histologically analyzed using Masson’s trichrome staining. Detection of organ toxicity was achieved using H&E staining. Measurements of the fibrosis ratio and left ventricular wall thickness were obtained using the Image J program. Immunohistochemical staining was performed to detect angiogenesis. Vessels were stained with CD31 antibody (Servicebio, China, GB12063) (1:500). Quantification of vascularization was performed using ImageJ software.

### 4.15 Statistical Analysis

Data were represented as the mean ± SD. Analytical procedures were done using Prism 8 (GraphPad) and SPSS 25.0. Independent *t* tests were used to compare two groups; one-way ANOVA with Tukey’s post hoc for intergroup differences. Non-normally distributed data were analyzed with the Mann–Whitney *U* test for two groups and Kruskal–Wallis with Dunn’s post hoc for multiple groups. The significance level was set at *P* < 0.05.

## 5 Limitation

In this study, the evaluation of cardiac function was limited to 28 days after administration of the EGCG@MPDA/CS hydrogel. The long-term impacts of the hydrogel will be investigated in future studies.

## Supporting Information Available

Cell compatibility testing of CS, MPDA/CS, and EGCG@MPDA/CS hydrogels; live/dead cell staining of H9C2 cells cultured with the 2 mg/mL EGCG@MPDA/CS hydrogel, 2 mg/mL MPDA/CS hydrogel, and CS hydrogel for 7 days; hemolysis assessment for the 2 mg/mL EGCG@MPDA/CS hydrogel, 2 mg/mL MPDA/CS hydrogel, and CS hydrogel; frequency sweep, compression test, and adhesion test of the hydrogels; in vitro degradation of the hydrogels; representative Western blot images and quantitative analysis of the partial reversal of the antiapoptotic effect of the EGCG@MPDA/CS hydrogel by the Nrf2 inhibitor ML385; schematic diagram of model construction and drug delivery; leukocyte levels among the hydrogel groups at 1, 3, and 7 days after MI treatment; and H&E staining of key organs (heart, spleen, liver, kidney, and lung) of rats in five groups (PDF)

## References

[ref1] Frampton J.; Devries J. T.; Welch T. D.; Gersh B. J. Modern Management of ST-Segment Elevation Myocardial Infarction. Curr. Probl. Cardiol. 2020, 45, 100393 10.1016/j.cpcardiol.2018.08.005. 30660333

[ref2] Hashimoto H.; Olson E. N.; Bassel-Duby R. Therapeutic approaches for cardiac regeneration and repair. Nat. Rev. Cardiol 2018, 15, 585–600. 10.1038/s41569-018-0036-6. 29872165 PMC6241533

[ref3] Kittleson M. M.; Kobashigawa J. A. Cardiac Transplantation: Current Outcomes and Contemporary Controversies. JACC Heart Fail 2017, 5, 857–868. 10.1016/j.jchf.2017.08.021. 29191293

[ref4] Yao C.; Veleva T.; Scott L.; Cao S.; Li L.; Chen G.; Jeyabal P.; Pan X.; Alsina K. M.; Abu-Taha I. D.; Ghezelbash S.; Reynolds C. L.; Shen Y. H.; LeMaire S. A.; Schmitz W.; Müller F. U.; El-Armouche A.; Tony Eissa N.; Beeton C.; Nattel S.; Wehrens X. H. T.; Dobrev D.; Li N. Enhanced Cardiomyocyte NLRP3 Inflammasome Signaling Promotes Atrial Fibrillation. Circulation 2018, 138, 2227–2242. 10.1161/CIRCULATIONAHA.118.035202. 29802206 PMC6252285

[ref5] Abbate A.; Toldo S.; Marchetti C.; Kron J.; Van Tassell B. W.; Dinarello C. A. Interleukin-1 and the Inflammasome as Therapeutic Targets in Cardiovascular Disease. Circ. Res. 2020, 126, 1260–1280. 10.1161/CIRCRESAHA.120.315937. 32324502 PMC8760628

[ref6] Grintzalis K.; Vernardis S. I.; Klapa M. I.; Georgiou C. D. Role of oxidative stress in Sclerotial differentiation and aflatoxin B1 biosynthesis in Aspergillus flavus. Appl. Environ. Microbiol. 2014, 80, 5561–5571. 10.1128/AEM.01282-14. 25002424 PMC4178614

[ref7] Fountain J. C.; Bajaj P.; Pandey M.; Nayak S. N.; Yang L.; Kumar V.; Jayale A. S.; Chitikineni A.; Zhuang W.; Scully B. T.; Lee R. D.; Kemerait R. C.; Varshney R. K.; Guo B. Oxidative stress and carbon metabolism influence Aspergillus flavus transcriptome composition and secondary metabolite production. Sci. Rep. 2016, 6, 38747 10.1038/srep38747. 27941917 PMC5150527

[ref8] Liu X.; Chen B.; Chen J.; Wang X.; Dai X.; Li Y.; Zhou H.; Wu L. M.; Liu Z.; Yang Y. A Cardiac-Targeted Nanozyme Interrupts the Inflammation-Free Radical Cycle in Myocardial Infarction. Adv. Mater. 2024, 36, e2308477 10.1002/adma.202308477. 37985164

[ref9] Zhang L.; Bei Z.; Li T.; Qian Z. An injectable conductive hydrogel with dual responsive release of rosmarinic acid improves cardiac function and promotes repair after myocardial infarction. Bioact Mater. 2023, 29, 132–150. 10.1016/j.bioactmat.2023.07.007. 37621769 PMC10444974

[ref10] Loureiro J.; Miguel S. P.; Galván-Chacón V.; Patrocinio D.; Pagador J. B.; Sánchez-Margallo F. M.; Ribeiro M. P.; Coutinho P. Three-Dimensionally Printed Hydrogel Cardiac Patch for Infarct Regeneration Based on Natural Polysaccharides. Polymers 2023, 15, 2824 10.3390/polym15132824. 37447470 PMC10346776

[ref11] Wu Z.; Li W.; Cheng S.; Liu J.; Wang S. Novel fabrication of bioengineered injectable chitosan hydrogel loaded with conductive nanoparticles to improve therapeutic potential of mesenchymal stem cells in functional recovery after ischemic myocardial infarction. Nanomedicine 2023, 47, 102616 10.1016/j.nano.2022.102616. 36374915

[ref12] M Ways T.; Lau W. M.; Khutoryanskiy V. V. Chitosan and Its Derivatives for Application in Mucoadhesive Drug Delivery Systems. Polymers 2018, 10, 267 10.3390/polym10030267. 30966302 PMC6414903

[ref13] Do N. H. N.; Truong Q. T.; Le P. K.; Ha A. C. Recent developments in chitosan hydrogels carrying natural bioactive compounds. Carbohydr. Polym. 2022, 294, 119726 10.1016/j.carbpol.2022.119726. 35868739

[ref14] Ma W. J.; Zhang X. X.; Liu Y. X.; Fan L.; Gan J. J.; Liu W. L.; Zhao Y. J.; Sun L. Y. Polydopamine Decorated Microneedles with Fe-MSC-Derived Nanovesicles Encapsulation for Wound Healing. Adv. Sci. 2022, 9, 12 10.1002/advs.202103317. PMC9069192 35266637

[ref15] Zhou L.; Dai C.; Fan L.; Jiang Y. H.; Liu C.; Zhou Z. N.; Guan P. F.; Tian Y.; Xing J.; Li X. J.; Luo Y. A.; Yu P.; Ning C. Y.; Tan G. X. Injectable Self-Healing Natural Biopolymer-Based Hydrogel Adhesive with Thermoresponsive Reversible Adhesion for Minimally Invasive Surgery. Adv. Funct. Mater. 2021, 31, 13 10.1002/adfm.202007457.

[ref16] Fang Z.; Lv Y.; Zhang H.; He Y.; Gao H.; Chen C.; Wang D.; Chen P.; Tang S.; Li J.; Qiu Z.; Shi X.; Chen L.; Yang J.; Chen X. A multifunctional hydrogel loaded with two nanoagents improves the pathological microenvironment associated with radiation combined with skin wounds. Acta Biomater. 2023, 159, 111–127. 10.1016/j.actbio.2023.01.052. 36736645

[ref17] Almatroodi S. A.; Almatroudi A.; Khan A. A.; Alhumaydhi F. A.; Alsahli M. A.; Rahmani A. H. Potential Therapeutic Targets of Epigallocatechin Gallate (EGCG), the Most Abundant Catechin in Green Tea, and Its Role in the Therapy of Various Types of Cancer. Molecules 2020, 25, 3146 10.3390/molecules25143146. 32660101 PMC7397003

[ref18] Bao X.; Zhao J.; Sun J.; Hu M.; Yang X. Polydopamine Nanoparticles as Efficient Scavengers for Reactive Oxygen Species in Periodontal Disease. ACS Nano 2018, 12, 8882–8892. 10.1021/acsnano.8b04022. 30028940

[ref19] Yang J.; Wang M.; Zheng S.; Huang R.; Wen G.; Zhou P.; Wang W.; Zhou S.; Jiang X.; Liu S.; Li Z.; Ma D.; Jiao G. Mesoporous polydopamine delivering 8-gingerol for the target and synergistic treatment to the spinal cord injury. J. Nanobiotechnology 2023, 21, 192 10.1186/s12951-023-01896-1. 37316835 PMC10268369

[ref20] Zhao W.; Liu Z.; Liang X.; Wang S.; Ding J.; Li Z.; Wang L.; Jiang Y. Preparation and characterization of epigallocatechin-3-gallate loaded melanin nanocomposite (EGCG @MNPs) for improved thermal stability, antioxidant and antibacterial activity. LWT 2022, 154, 112599 10.1016/j.lwt.2021.112599.

[ref21] Quan T. H.; Benjakul S. Duck egg albumen hydrolysate-epigallocatechin gallate conjugates: Antioxidant, emulsifying properties and their use in fish oil emulsion. Colloid Surf. A-Physicochem. Eng. Asp. 2019, 579, 123711 10.1016/j.colsurfa.2019.123711.

[ref22] Poinard B.; Neo S. Z. Y.; Yeo E. L. L.; Heng H. P. S.; Neoh K. G.; Kah J. C. Y. Polydopamine Nanoparticles Enhance Drug Release for Combined Photodynamic and Photothermal Therapy. ACS Appl. Mater. Interfaces 2018, 10, 21125–21136. 10.1021/acsami.8b04799. 29871485

[ref23] Saravanan S.; Vimalraj S.; Thanikaivelan P.; Banudevi S.; Manivasagam G. A review on injectable chitosan/beta glycerophosphate hydrogels for bone tissue regeneration. Int. J. Biol. Macromol. 2019, 121, 38–54. 10.1016/j.ijbiomac.2018.10.014. 30291931

[ref24] Roy S.; Kim H. C.; Kim J. W.; Zhai L.; Zhu Q. Y.; Kim J. Incorporation of melanin nanoparticles improves UV-shielding, mechanical and antioxidant properties of cellulose nanofiber based nanocomposite films. Mater. Today Commun. 2020, 24, 100984 10.1016/j.mtcomm.2020.100984.

[ref25] Zeng J.; Ren X.; Zhu S.; Gao Y. Fabrication and characterization of an economical active packaging film based on chitosan incorporated with pomegranate peel. Int. J. Biol. Macromol. 2021, 192, 1160–1168. 10.1016/j.ijbiomac.2021.10.064. 34678378

[ref26] Bao R.; Tan B.; Liang S.; Zhang N.; Wang W.; Liu W. A π-π conjugation-containing soft and conductive injectable polymer hydrogel highly efficiently rebuilds cardiac function after myocardial infarction. Biomaterials 2017, 122, 63–71. 10.1016/j.biomaterials.2017.01.012. 28107665

[ref27] Wang W.; Chen J.; Li M.; Jia H.; Han X.; Zhang J.; Zou Y.; Tan B.; Liang W.; Shang Y.; Xu Q.; A S.; Wang W.; Mao J.; Gao X.; Fan G.; Liu W. Rebuilding Postinfarcted Cardiac Functions by Injecting TIIA@PDA Nanoparticle-Cross-linked ROS-Sensitive Hydrogels. ACS Appl. Mater. Interfaces 2019, 11, 2880–2890. 10.1021/acsami.8b20158. 30592403

[ref28] Qin Y.; Liu Y.; Yuan L.; Yong H.; Liu J. Preparation and characterization of antioxidant, antimicrobial and pH-sensitive films based on chitosan, silver nanoparticles and purple corn extract. Food Hydrocolloids 2019, 96, 102–111. 10.1016/j.foodhyd.2019.05.017.

[ref29] Zagury Y.; Kazir M.; Livney Y. D. Improved antioxidant activity, bioaccessibility and bioavailability of EGCG by delivery in β-lactoglobulin particles. Journal of Functional Foods 2019, 52, 121–130. 10.1016/j.jff.2018.10.025.

[ref30] Wang D.; Hu Y.; Zhang L.; Cai H.; Wang Y.; Zhang Y. Dual delivery of an NF-κB inhibitor and IL-10 through supramolecular hydrogels polarizes macrophages and promotes cardiac repair after myocardial infarction. Acta Biomater. 2023, 164, 111–123. 10.1016/j.actbio.2023.03.035. 37001840

[ref31] Chen L.; Li S.; Zhu J.; You A.; Huang X.; Yi X.; Xue M. Mangiferin prevents myocardial infarction-induced apoptosis and heart failure in mice by activating the Sirt1/FoxO3a pathway. J. Cell. Mol. Med. 2021, 25, 2944–2955. 10.1111/jcmm.16329. 33523605 PMC7957271

[ref32] Zhang Q.; Wang L.; Wang S.; Cheng H.; Xu L.; Pei G.; Wang Y.; Fu C.; Jiang Y.; He C.; Wei Q. Signaling pathways and targeted therapy for myocardial infarction. Signal Transduct Target Ther 2022, 7, 78 10.1038/s41392-022-00925-z. 35273164 PMC8913803

[ref33] Wang B.; Wu C. F.; He S. F.; Wang Y. G.; Wang D.; Tao H.; Wang C. C.; Pang X. X.; Li F.; Yuan Y.; Gross E. R.; Liang G. L.; Zhang Y. V1-Cal hydrogelation enhances its effects on ventricular remodeling reduction and cardiac function improvement post myocardial infarction. Chem. Eng. J. 2022, 433, 134450 10.1016/j.cej.2021.134450. 36338580 PMC9634955

[ref34] Broughton K. M.; Wang B. J.; Firouzi F.; Khalafalla F.; Dimmeler S.; Fernandez-Aviles F.; Sussman M. A. Mechanisms of Cardiac Repair and Regeneration. Circ. Res. 2018, 122, 1151–1163. 10.1161/CIRCRESAHA.117.312586. 29650632 PMC6191043

[ref35] Wang X. W.; Guo Z. K.; Ding Z. F.; Mehta J. L. Inflammation, Autophagy, and Apoptosis After Myocardial Infarction. J. Am. Heart Assoc. 2018, 7, 13 10.1161/jaha.117.008024. PMC6015297 29680826

[ref36] Frangogiannis N. G. Inflammation in cardiac injury, repair and regeneration. Curr. Opin Cardiol 2015, 30, 240–245. 10.1097/HCO.0000000000000158. 25807226 PMC4401066

[ref37] Oliveira J. B.; Soares A.; Sposito A. C. Inflammatory Response During Myocardial Infarction. Adv. Clin Chem. 2018, 84, 39–79. 10.1016/bs.acc.2017.12.002. 29478516

[ref38] Zhou S. F.; Yuan J.; Liao M. Y.; Xia N.; Tang T. T.; Li J. J.; Jiao J.; Dong W. Y.; Nie S. F.; Zhu Z. F.; Zhang W. C.; Lv B. J.; Xiao H.; Wang Q.; Tu X.; Liao Y. H.; Shi G. P.; Cheng X. IL-17A promotes ventricular remodeling after myocardial infarction. J. Mol. Med. (Berl) 2014, 92, 1105–1116. 10.1007/s00109-014-1176-8. 24965614

[ref39] Hao T.; Qian M.; Zhang Y.; Liu Q.; Midgley A. C.; Liu Y.; Che Y.; Hou J.; Zhao Q. An Injectable Dual-Function Hydrogel Protects Against Myocardial Ischemia/Reperfusion Injury by Modulating ROS/NO Disequilibrium. Adv. Sci. (Weinh) 2022, 9, e2105408 10.1002/advs.202105408. 35319828 PMC9130918

[ref40] Kim Y. S.; Jeong H. Y.; Kim A. R.; Kim W. H.; Cho H.; Um J.; Seo Y.; Kang W. S.; Jin S. W.; Kim M. C.; Kim Y. C.; Jung D. W.; Williams D. R.; Ahn Y. Natural product derivative BIO promotes recovery after myocardial infarction via unique modulation of the cardiac microenvironment. Sci. Rep. 2016, 6, 30726 10.1038/srep30726. 27510556 PMC4980696

[ref41] Fan C.; Shi J.; Zhuang Y.; Zhang L.; Huang L.; Yang W.; Chen B.; Chen Y.; Xiao Z.; Shen H.; Zhao Y.; Dai J. Myocardial-Infarction-Responsive Smart Hydrogels Targeting Matrix Metalloproteinase for On-Demand Growth Factor Delivery. Adv. Mater. 2019, 31, e1902900 10.1002/adma.201902900. 31408234

[ref42] Michelis K. C.; Boehm M.; Kovacic J. C. New vessel formation in the context of cardiomyocyte regeneration--the role and importance of an adequate perfusing vasculature. Stem Cell Res. 2014, 13, 666–682. 10.1016/j.scr.2014.04.009. 24841067 PMC4213356

[ref43] Meng J.; Chen Y.; Wang J.; Qiu J.; Chang C.; Bi F.; Wu X.; Liu W. EGCG protects vascular endothelial cells from oxidative stress-induced damage by targeting the autophagy-dependent PI3K-AKT-mTOR pathway. Ann. Transl Med. 2020, 8, 200 10.21037/atm.2020.01.92. 32309347 PMC7154459

[ref44] Shi J.; Zhang M.; Zhang L.; Deng H. Epigallocatechin-3-gallate attenuates microcystin-LR-induced apoptosis in human umbilical vein endothelial cells through activation of the NRF2/HO-1 pathway. Environ. Pollut. 2018, 239, 466–472. 10.1016/j.envpol.2018.04.038. 29679944

[ref45] Yin J.; Huang F.; Yi Y.; Yin L.; Peng D. EGCG attenuates atherosclerosis through the Jagged-1/Notch pathway. Int. J. Mol. Med. 2016, 37, 398–406. 10.3892/ijmm.2015.2422. 26648562

[ref46] Bhatt A. S.; Ambrosy A. P.; Velazquez E. J. Adverse Remodeling and Reverse Remodeling After Myocardial Infarction. Curr. Cardiol Rep 2017, 19, 71 10.1007/s11886-017-0876-4. 28660552

[ref47] Zhou Y.; Xu B.; Zhou P.; Chen X.; Jiao G.; Li H. Gold@mesoporous polydopamine nanoparticles modified self-healing hydrogel for sport-injuring therapy. Int. J. Biol. Macromol. 2023, 253, 127441 10.1016/j.ijbiomac.2023.127441. 37839604

[ref48] Liu Z.; Wang H.; Wang Y.; Lin Q.; Yao A.; Cao F.; Li D.; Zhou J.; Duan C.; Du Z.; Wang Y.; Wang C. The influence of chitosan hydrogel on stem cell engraftment, survival and homing in the ischemic myocardial microenvironment. Biomaterials 2012, 33, 3093–3106. 10.1016/j.biomaterials.2011.12.044. 22265788

[ref49] Jia Q.; Fu Z.; Li Y.; Kang Z.; Wu Y.; Ru Z.; Peng Y.; Huang Y.; Luo Y.; Li W.; et al. Hydrogel Loaded with Peptide-Containing Nanocomplexes: Symphonic Cooperation of Photothermal Antimicrobial Nanoparticles and Prohealing Peptides for the Treatment of Infected Wounds. ACS Applied Materials And Interfaces 2024, 16, 13422–13438. 10.1021/acsami.3c16061. 38442213

[ref50] Fu Z.; Sun H.; Wu Y.; Li C.; Wang Y.; Liu Y.; Li Y.; Nie J.; Sun D.; Zhang Y.; Liu N.; Guo K.; Yin S.; Jia Q.; Yang Y.; He L.; Wang Y.; Yang X. A cyclic heptapeptide-based hydrogel boosts the healing of chronic skin wounds in diabetic mice and patients. NPG Asia Mater. 2022, 14, 99 10.1038/s41427-022-00444-x.

[ref51] Xia W.; Lai G.; Li Y.; Zeng C.; Sun C.; Zhang P.; Zhu G.; Li L.; Wu L. Photo-crosslinked adhesive hydrogel loaded with extracellular vesicles promoting hemostasis and liver regeneration. Front Bioeng Biotechnol 2023, 11, 1170212 10.3389/fbioe.2023.1170212. 37234477 PMC10208220

[ref52] Zarkesh I.; Movahedi F.; Sadeghi-Abandansari H.; Pahlavan S.; Soleimani M.; Baharvand H. ROS scavenging activity of polydopamine nanoparticle-loaded supramolecular gelatin-based hydrogel promoted cardiomyocyte proliferation. Int. J. Biol. Macromol. 2024, 259, 129228 10.1016/j.ijbiomac.2024.129228. 38184051

[ref53] Zhao W.; Liang X.; Wang X.; Wang S.; Wang L.; Jiang Y. Chitosan based film reinforced with EGCG loaded melanin-like nanocomposite (EGCG@MNPs) for active food packaging. Carbohydr. Polym. 2022, 290, 119471 10.1016/j.carbpol.2022.119471. 35550766

[ref54] Hao T.; Li J.; Yao F.; Dong D.; Wang Y.; Yang B.; Wang C. Injectable Fullerenol/Alginate Hydrogel for Suppression of Oxidative Stress Damage in Brown Adipose-Derived Stem Cells and Cardiac Repair. ACS Nano 2017, 11, 5474–5488. 10.1021/acsnano.7b00221. 28590722

[ref55] Zhou J.; Liu W.; Zhao X.; Xian Y.; Wu W.; Zhang X.; Zhao N.; Xu F. J.; Wang C. Natural Melanin/Alginate Hydrogels Achieve Cardiac Repair through ROS Scavenging and Macrophage Polarization. Adv. Sci. (Weinh) 2021, 8, e2100505 10.1002/advs.202100505. 34414693 PMC8529445

[ref56] Yuan Z.; Tsou Y. H.; Zhang X. Q.; Huang S.; Yang Y.; Gao M.; Ho W.; Zhao Q.; Ye X.; Xu X. Injectable Citrate-Based Hydrogel as an Angiogenic Biomaterial Improves Cardiac Repair after Myocardial Infarction. ACS Appl. Mater. Interfaces 2019, 11, 38429–38439. 10.1021/acsami.9b12043. 31573790

