# Supplementary material for: Drug-Loaded Mesoporous Polydopamine Nanoparticles in Chitosan Hydrogels Enable Myocardial Infarction Repair through ROS Scavenging and Inhibition of Apoptosis
Source: ACS Appl Mater Interfaces. 2024 Sep 30;16(45):61551–64. doi: 10.1021/acsami.4c08155 (PMC11566824; doi:10.1021/acsami.4c08155)
Supplement: Supplementary file 1 — am4c08155_si_001.pdf [file am4c08155_si_001.pdf]

Supporting Information

Drug-loaded Mesoporous Polydopamine Nanoparticles in Chitosan Hydrogel Enable Myocardial Infarction Repair through ROS Scavenging and Inhibition of Apoptosis

Tianhu Wang[1], Yabin Wang[1], Yingjie Zhang[1], Zhiyi Fang[2], Sulei Li[1], Zhenghui Gu[1], Yan Ma[1], Linghuan Wang[2], Dong Han[1], Changyong Wang[3], Jin Zhou[3] and Feng Cao[1]*

[1] Chinese PLA Medical School & Department of Cardiology, The Second Medical Center , National Clinical Research Center for Geriatric Diseases, Chinese PLA General Hospital, Beijing 100853, China.

[2] School of Medicine, Nankai University, Tianjin 300071, China.

[3] Beijing Institute of Basic Medical Sciences, Beijing 100850, China.

* Corresponding author to fengcao8828@163.com.

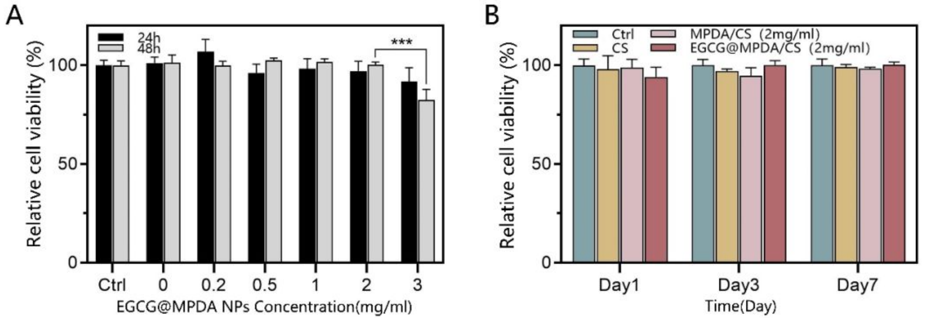

Figure S1 Cell compatibility testing of hydrogels. (A) The effect of EGCG@MPDA/CS hydrogels with different concentrations of EGCG@MPDA NPs on the viability of H9C2 cells. (B) The effect of CS hydrogels, MPDA/CS hydrogels (2mg/ml), and EGCG@MPDA/CS (2mg/ml) hydrogels on the viability of H9C2 cells. (Results are presented as mean ± SD. n=6 for each group, $^{***}P < 0.001$)

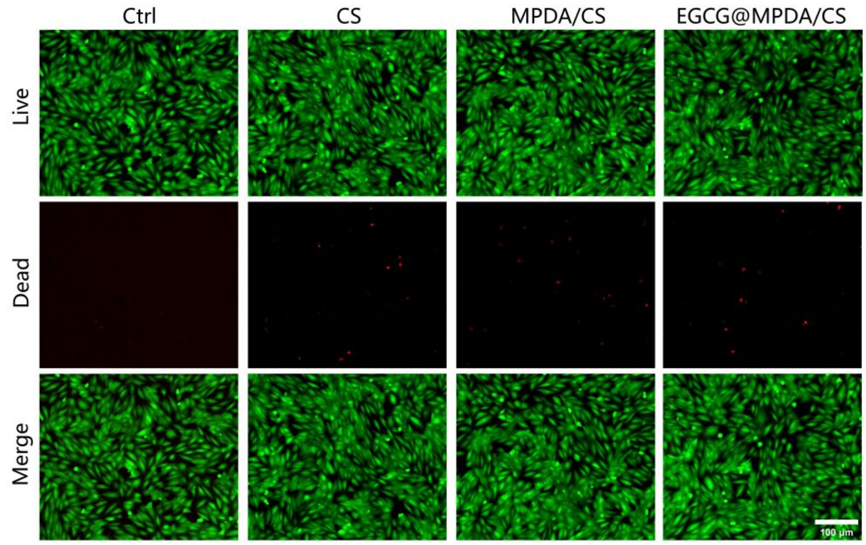

Figure S2 Live/dead cell staining of H9C2 cells after 7 days of culture with extracts from CS hydrogels, MPDA/CS hydrogels (2mg/ml), and EGCG@MPDA/CS (2mg/ml) hydrogels.

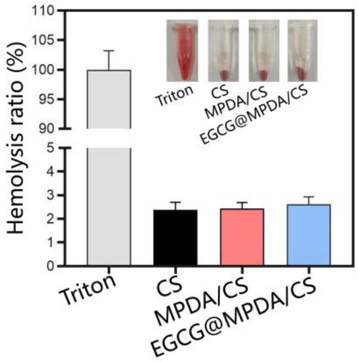

Figure S3 Hemolysis assessment for the 2 mg/ml EGCG@MPDA/CS hydrogel, 2 mg/ml MPDA/CS hydrogel, and CS hydrogel. (Results are presented as mean ± SD. n = 3 for each group)

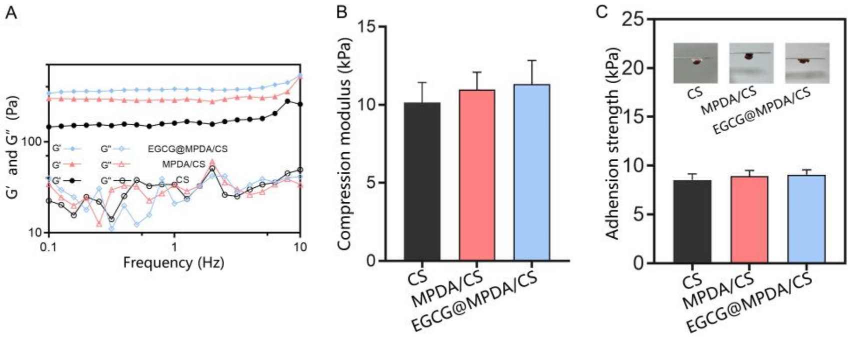

Figure S4 Frequency sweep, compression test, and adhesion test of the hydrogels. (A) Frequency sweep of the hydrogels. (B) Adhesion strengths of the hydrogels. (n = 3 for each group). (C) Compression strengths of the hydrogels (n = 5 for each group). (Results are presented as mean ± SD)

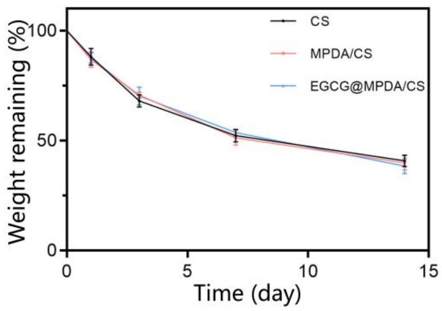

Figure S5 *In vitro* degradation of the hydrogels. (Results are presented as mean ± SD. n=3 for each group)

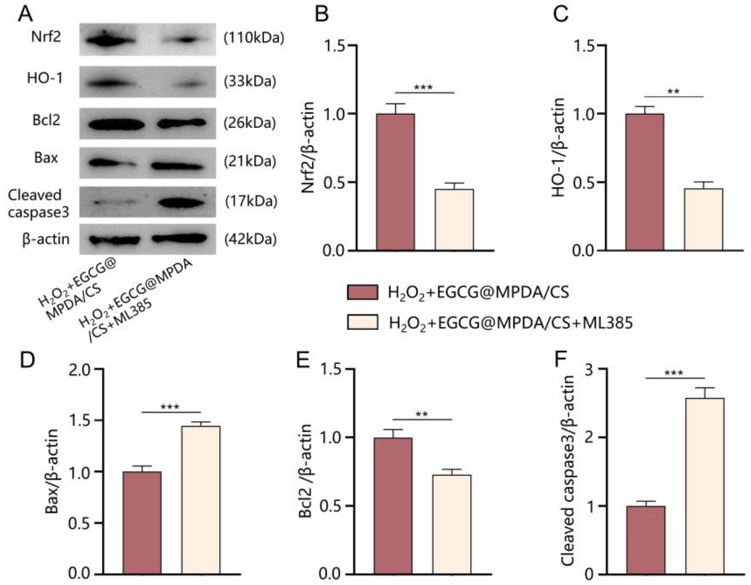

Figure S6 Representative Western blot images and quantitative analysis of the partial reversal of the anti-apoptotic effect of EGCG@MPDA/CS hydrogel by the Nrf2 inhibitor ML385. (A) Representative western blot images of Nrf2, HO-1, Bcl2, Bax, Cleaved caspase3, and β-actin. Quantification analysis of Bax (B), Bcl2 (C), Cleaved caspase3 (D), Nrf2 (E), and HO-1 (F). (Results are presented as mean ± SD. n=3 for each group. $^{**}P < 0.01$, $^{***}P < 0.001$)

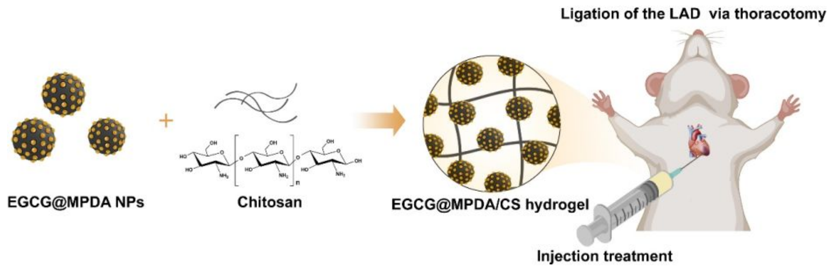

Figure S7 The schematic diagram of model construction and drug delivery.

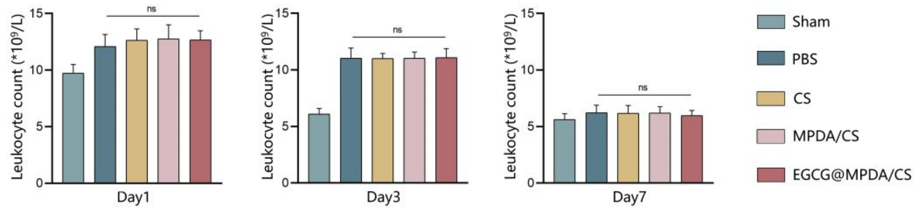

Figure S8 The leukocyte levels among the hydrogel groups at 1, 3, and 7 days post MI treatment. (Results are presented as mean ± SD. n=3 for each group, $^{ns}P > 0.05$)

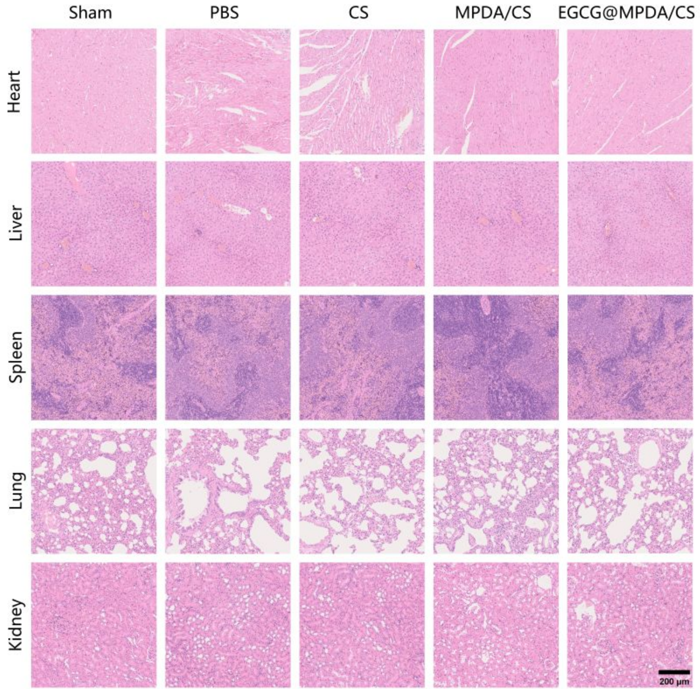

Figure S9 H&E staining of main organs (heart, liver, spleen, lung, and kidney) of rats in five groups.
